# Roles of airway and intestinal epithelia in responding to pathogens and maintaining tissue homeostasis

**DOI:** 10.3389/fcimb.2024.1346087

**Published:** 2024-04-26

**Authors:** Marcela Ambrogi, Chad M. Vezina

**Affiliations:** Department of Comparative Biosciences, School of Veterinary Medicine, University of Wisconsin-Madison, Madison, WI, United States

**Keywords:** epithelial homeostasis, pathogen detection, immune signaling, tissue repair, barrier integrity

## Abstract

Epithelial cells form a resilient barrier and orchestrate defensive and reparative mechanisms to maintain tissue stability. This review focuses on gut and airway epithelia, which are positioned where the body interfaces with the outside world. We review the many signaling pathways and mechanisms by which epithelial cells at the interface respond to invading pathogens to mount an innate immune response and initiate adaptive immunity and communicate with other cells, including resident microbiota, to heal damaged tissue and maintain homeostasis. We compare and contrast how airway and gut epithelial cells detect pathogens, release antimicrobial effectors, collaborate with macrophages, Tregs and epithelial stem cells to mount an immune response and orchestrate tissue repair. We also describe advanced research models for studying epithelial communication and behaviors during inflammation, tissue injury and disease.

## Introduction

The biology of epithelial cells positioned where the body interfaces with the external environment is unique in that these cells are the first line of defense against a barrage of potential threats. The epithelial cells at the interface possess a sophisticated array of receptors, including pattern recognition receptors (PRRs) situated on their outer membrane and within the cytosol. This sophisticated system enables epithelial cells to detect invading pathogens, environmental toxins, and signs of tissue damage. Sensors such as the intracellular aryl hydrocarbon receptor (AHR) and membrane-bound toll-like receptors (TLRs) allow epithelial cells to perceive environmental factors like dietary metabolites and allergens. Furthermore, epithelial cells actively surveil for barrier breaches, such as the loss of intercellular junctions that arise during tissue damage (Larsen et al., 2020; Zheng et al., 2020b), and respond with finely tuned defensive and reparative mechanisms to maintain homeostasis (Larsen et al., 2020).

The epithelium of the gastrointestinal, respiratory, skin, and urogenital tracts, guard the body from the outside world by forming a dynamic and immunologically active barrier, capable of sensing environmental changes and engaging with resident and recruited immune cells to mount a robust defense (Pott and Hornef, 2012). The collaborative reactions of epithelial, stromal, and immune cells within these unique tissue microenvironments shape the body’s first line of defense (Larsen et al., 2020; Hewitt and Lloyd, 2021).

Epithelial tissue is structured by a close packing of cells, cells which are typically polarized and harbor distinct apical, basal, and lateral surfaces. Epithelial cell polarity facilities essential functions such as absorption and secretion and establishes a protective defense barrier. Epithelial cells are held together by various junctions including tight junctions (TJ), adherens junctions (AJ), and desmosomes to maintain tissue integrity (Peterson and Artis, 2014).

Epithelial tissues regulate nutrient and water passage, establish an entry barrier against pathogens and facilitate gas exchange. Whether it is the epidermis guarding against environmental toxins, the gastrointestinal epithelium absorbing nutrients, the urethral epithelium creating a barrier for urine, or the respiratory epithelium filtering and humidifying inhaled air, epithelial tissues near the interface with the outside of the body are indispensable for maintaining homeostasis and protecting the body from harm (Pott and Hornef, 2012; Zhang and Atala, 2013; Larsen et al., 2020).

In this review, we specifically delve into the intricate interplay of immune signaling pathways and epithelial homeostasis, with a particular emphasis on airway and gut epithelium. By shedding light on the significant roles of these epithelial tissues in protecting against pathogens during both health and disease, we aim to provide a more comprehensive understanding of the innate immunity offered by the epithelium in these critical anatomical locations.

### The epithelial realm

Epithelial tissues can be organized in many ways, but share fundamental characteristics (Giancotti and Tarone, 2003; Blanpain et al., 2007). The developing epithelium begins as a sheet of cells firmly adhered to the basement membrane. The basement membrane is rich in extracellular matrix and growth factors and forms the boundary between the epithelium and the underlying mesenchyme. The epithelium is vital for orchestrating organogenesis (Fuchs and Raghavan, 2002; Blanpain et al., 2006), and secretes or responds to diverse developmental signaling factors including Sonic hedgehog (SHH), Notch and Wingless-activated (WNT) proteins (Jeng et al., 2020).

**Figure 1:**
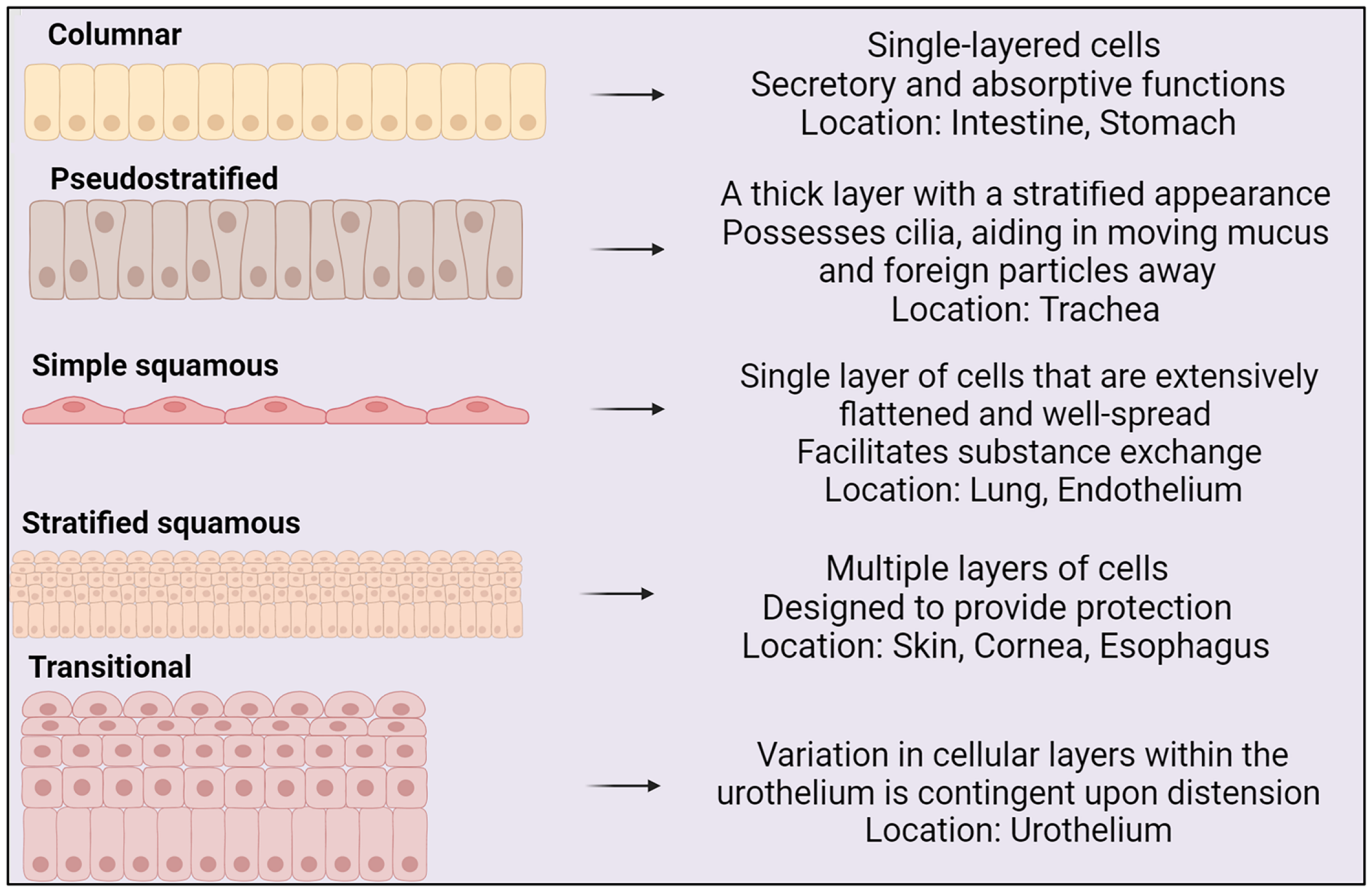
Types of epithelium, their structures and locations within the body (BioRender, 2023).

The shape and structure of epithelial cells aligns with cellular function (see **Figure 1**) (Vrana et al., 2013). Epithelial cells express transmembrane integrin heterodimers, connecting them through collagen to the extracellular matrix. Integrins link to the cellular cytoskeleton and are instrumental in cell migration, stratification, and differentiation (Walko et al., 2015).

Epithelial focal adhesions and hemidesmosomes act as gatekeepers, enabling cells to build and remodel connections with the extracellular matrix. Through adherens junctions, tight junctions, and desmosomes, epithelial cells establish vital intercellular connections. These connections enable epithelial cells to communicate seamlessly and function as a unified sheet (Fuchs and Raghavan, 2002; Shin et al., 2006).

Epithelial tissues rely on a continuous supply of new cells for self-renewal and tissue homeostasis, which are primarily governed by progenitor cells. The epithelium is built from transient populations of organ-specific progenitor cells residing in epithelial or mesenchymal compartments (Bjerknes and Cheng, 1999). Progenitor cells are essential for tissue development, maintenance, and repair. While progenitor cells have varying degrees of lineage commitment, their self-renewal capacity is typically more limited than that of stem cells, which continuously divide to produce more stem cells (Bjerknes and Cheng, 1999; Blanpain et al., 2007; Rawlins, 2008). This perpetual cycle of cellular regeneration ensures epithelial structures remain robust and operational over their lifetimes (Blanpain et al., 2007; Blanpain and Fuchs, 2009).

#### Gut epithelium: organization and function

In the small intestine, the crypt-villus architecture defines functional zones. Villi are comprised of differentiated cells including enterocytes, goblet cells, enteroendocrine cells (EECs), and Paneth cells, which serve specific roles in nutrient absorption, mucus production, hormone release, and immune defense (Ayabe et al., 2000; Johansson et al., 2008). Hormones such as cholecystokinin (produced by enteroendocrine cells), secretin (produced by enteroendocrine S cells), Gastric Inhibitory Polypeptide (GIP) (produced by enteroendocrine K cells), and motilin (produced by enteroendocrine M cells) regulate various digestive processes and contribute to the coordination of nutrient absorption and gut motility (see **Table 1**) (Murphy and Bloom, 2006).

**Table 1:** Epithelial cell types and hormone secretion in the small and large intestines.

	Small intestine	Large intestine	Reference
Cell types			
Enterocyte	Present	Present	(Kayisoglu et al., 2021)
TA cell	Present	Present	(Umar, 2010; Kayisoglu et al., 2021)
Goblet cell	Present	Present	(Pelaseyed et al., 2014; Kayisoglu et al., 2021)
Enteroendocrine cell	Present	Present	(Kayisoglu et al., 2021; Rezzani et al., 2022)
Paneth cell	Present	Absent	(Ayabe et al., 2000; Kayisoglu et al., 2021)
Stem cell	Present	Present	(Umar, 2010; Pelaseyed et al., 2014; Kayisoglu et al., 2021)
Tuft cell	Present	Present	(Gerbe et al., 2012; Kayisoglu et al., 2021)
Microfold (M) cell	Present	Absent	(Kraehenbuhl and Neutra, 2000; Kayisoglu et al., 2021)
Deep secretory cell	Absent	Present	(Kayisoglu et al., 2021; Schumacher et al., 2023)
Hormones			
Cholecystokinin	Present	Absent	(Murphy and Bloom, 2006; Berna and Jensen, 2007)
Secretin	Present	Absent	(Fine et al., 1983; Murphy and Bloom, 2006)
Gastric Inhibitory Polypeptide	Present	Absent	(Murphy and Bloom, 2006; Iwasaki et al., 2015)
Motilin	Present	Absent	(Murphy and Bloom, 2006; De Smet et al., 2009)
Serotonin	Present	Absent	(Andreeva and Makarova, 2013)
Glucagon-like peptide 1	Low	Present	(Drucker, 1998; Murphy and Bloom, 2006; Chambers et al., 2015)
Glucagon-like peptide 2	Low	Present	(Drucker, 1998; Murphy and Bloom, 2006)
Oxyntomodulin	Low	Present	(Murphy and Bloom, 2006; Wewer Albrechtsen et al., 2016)
Peptide YY	Low	Present	(Murphy and Bloom, 2006; Chambers et al., 2015)

Active cell proliferation in crypts maintains stem cells, Paneth cells and transit amplifying cells (TA). In the small intestine, the mucus layer is thinner and firmly attached to the epithelium, ensuring selective nutrient transport (Umar, 2010; Pelaseyed et al., 2014). In contrast, the large intestine’s inner mucus layer acts as a protective barrier, effectively separating commensal bacteria from the host epithelium to minimize bacterial exposure. Concurrently, the outer mucus layer serves as a conducive environment, fostering the thriving presence of commensal bacteria, thus establishing their natural habitat (Pelaseyed et al., 2014).

In the expansive terrain of the large intestine, where the traditional villi structure is absent, tissue homeostasis and regeneration is controlled by colonic crypts, which harbor stem cells. Within the dynamic microenvironment of the crypt, colonocytes and goblet cells, the latter more abundant than in the small intestine, play distinct roles in nutrient absorption and mucus production, respectively (Pelaseyed et al., 2014). Enteroendocrine cells in the large intestine secrete Glucagon-Like Peptide 1 (GLP-1), Glucagon-Like Peptide 2 (GLP-2), oxyntomodulin, and Peptide YY (PYY) (Murphy and Bloom, 2006). The continuous renewal of colonic epithelium is orchestrated by colonic stem cells residing at the base of crypts. Through asymmetric cell division, colonic stem cells give rise to TA. TA cells undergo a finite number of divisions before terminally differentiating into various epithelial cell types, including colonocytes, EECs, tuft cells and goblet cells. The resilience of the stem cell population remains integral to sustaining cellular dynamics and overall tissue integrity (Velázquez et al., 1997; Litvak et al., 2018).

The large intestine harbors a substantial mucus layer, serving as a shield against resident microbiota (Shan et al., 2013). The mucous layer supports beneficial bacteria and acts as a barrier, impeding pathogen invasion and maintaining gut balance. The metabolic process of healthy colonocytes plays a pivotal role in preserving anaerobic conditions in the gut lumen, achieved through rapid oxygen consumption (Cummings et al., 1987). Such anaerobic conditions create a conducive environment for obligate anaerobic organisms, particularly those involved in the breakdown of dietary fiber, yielding short-chain fatty acids that benefit the host (Cummings et al., 1987; Litvak et al., 2018).

**Figure 2:**
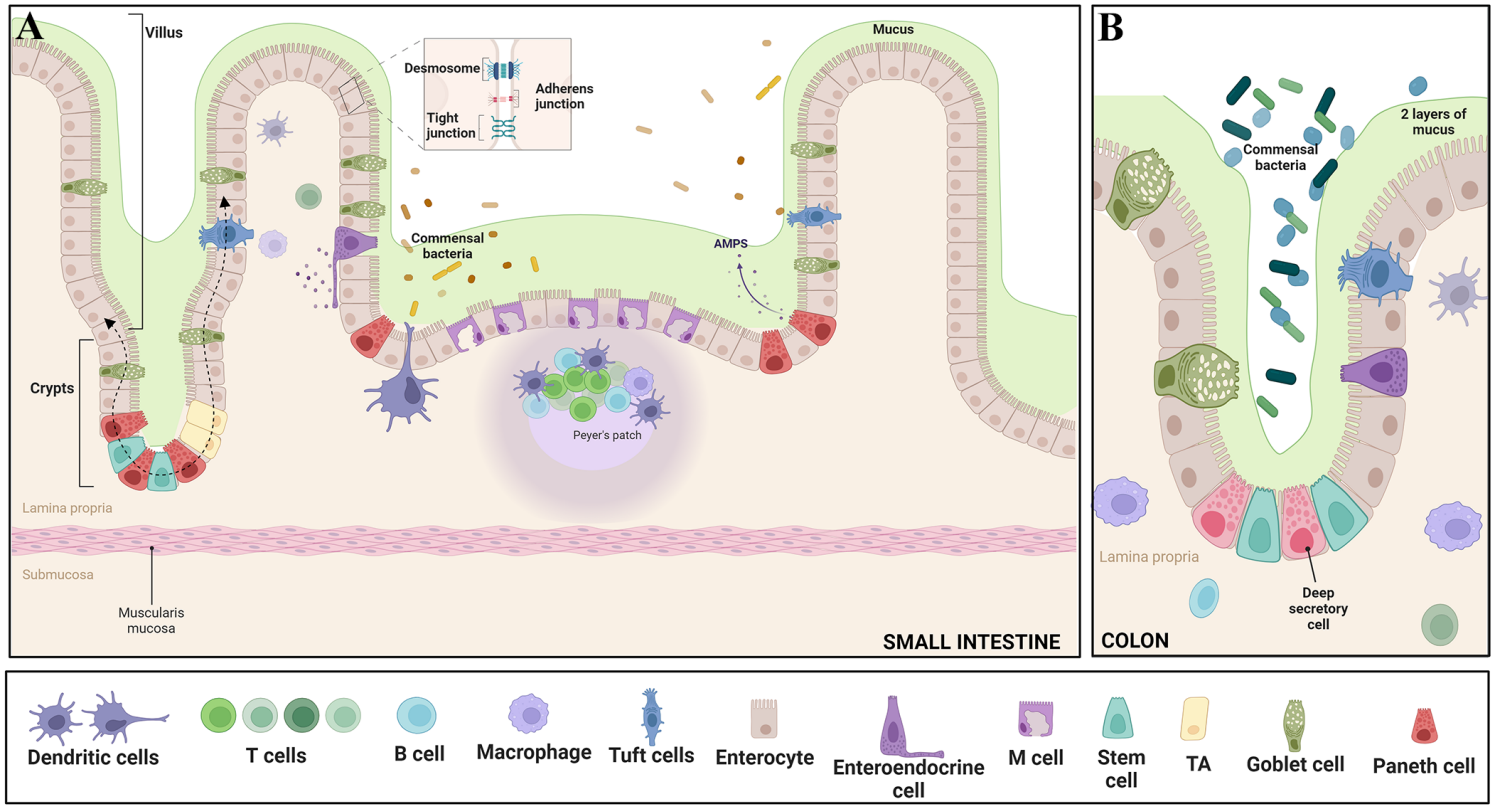
Epithelial features of the gut - The intricate intestinal epithelium harbors a variety of specialized cell types crucial for tissue function. Enterocytes play a key role in nutrient absorption, while Intestinal stem cells and Transit Amplifying Progenitors (TA) contribute to cellular proliferation. Goblet cells secrete protective mucus, and enteroendocrine cells release hormones regulating diverse gut functions. Paneth cells offer antimicrobial defense, tuft cells protect against parasites, and M cells assist in antigen presentation. Intestine-resident macrophages sample the luminal environment through transepithelial dendrites. Differentiated IECs, excluding Paneth cells, migrate upward along the crypt–villus axis, guided by dashed arrows. Moreover, the epithelium is reinforced by essential junctional complexes: Tight junctions seal intercellular spaces, Adherens junctions anchor neighboring cells, and Desmosomes provide structural integrity by connecting cell cytoskeletons. These diverse cell types and junctions collectively orchestrate proper intestinal function, encompassing nutrient absorption, immune response, and barrier integrity. Panel **(A)** illustrates the features in the small intestine, while panel **(B)** depicts the characteristics in the colon. Adapted from “Gut-Brain axis,” by BioRender.com (BioRender, 2023).

The intestinal epithelium harbors undifferentiated intestinal stem cells (ISCs), situated within crypts, which serve as progenitors for cellular regeneration and maintenance. ISCs can self-renew and give rise to various cell types. Asymmetric division of ISCs generates TA cells, rapidly dividing progenitors fueling intestinal cell turnover essential for gut homeostasis and repair (Cheng and Leblond, 1974; Schmidt et al., 1988). TA cells, which originate in the crypt, give rise to absorptive cells (enterocytes) or secretory lineages (goblet cells, tuft cell, enteroendocrine cell and paneth cells), as they migrate upward into the villus (**Figure 2**) (Umar, 2010; Spit et al., 2018). ISC and TA cell division and differentiation ensures timely renewal of the small intestinal epithelial lining every 4 to 5 days (Cheng and Leblond, 1974; Umar, 2010).

Absorptive enterocytes, the primary cells within the small intestine’s villi, specialize in nutrient absorption. Their distinctive brush border, adorned with microvilli supported by a cytoskeletal core and terminal web, significantly amplifies the surface area for digestive enzymes and transporters (Shen, 2009). Featuring an enzyme-rich apical membrane and a basolateral membrane geared for nutrient transport, enterocytes employ a dual membrane setup to optimize nutrient absorption (Jacob and Naim, 2001; Ang et al., 2004).

Enterocytes contribute to intestinal health by secreting antimicrobial proteins, supporting cellular processes like autophagy (Benjamin et al., 2013; Conway et al., 2013; Wlodarska et al., 2014). Autophagy plays a pivotal role in maintaining cellular homeostasis (Rabinowitz and White, 2010), and is essential for the recognition and degradation of intracellular pathogens, contributing to the innate response to pathogens (Deretic and Levine, 2009; Benjamin et al., 2013).

Enterocytes produce several factors to coordinate immune responses. These include chemokines that attract neutrophils (CXCL8, CXCL1, CXCL3, and CXCL5), macrophages and dendritic cells (CCL2), dendritic cells and memory T cells (CCL20), dendritic cells and Th2 cells (CCL22), Th1 cells (CXCL9, CXCL10, and CXCL11), plasma cells (CCL28), α4β7 T cells (CCL25, also known as TECK), and cytokines (GF-α, IL-1, IL-6, IL-10, IL-15, IL-18, GM-CSF, TGF-β, IL-8, MIP-3α) (Dwinell et al., 1999; Stadnyk, 2002; Kagnoff, 2014; Hooper, 2015). TNF-α is a pro-inflammatory cytokine produced by epithelial cells and immune cells in response to infection or injury. It promotes inflammation, activates immune cells, and contributes to tissue damage and repair. GM-CSF stimulates the production and function of immune cells, including macrophages and dendritic cells, enhancing the immune response against pathogens (Mahapatro et al., 2021; Kagnoff, 2014). Furthermore, enterocytes facilitate the transport of secretory immunoglobulin A from the basolateral to the apical surface, playing a vital role in maintaining homeostasis between host tissues and the intestinal microbial communities (Hooper, 2015).

Tuft cells are a major source of interleukin-25 (IL-25) in the intestine (von Moltke et al., 2016; Desai et al., 2021). Upon helminth infection, IL-25 produced by Tuft cells activates group 2 innate lymphoid cells (ILC2) to secrete IL-13, which initiates an immune response against parasites. Tuft cells play a critical role in immunity against parasite infection, but whether Tuft cells participate in bacterial infection is unclear (Xiong et al., 2022). Tuft cells derive from ISCs, under steady conditions and in response to injury (von Moltke et al., 2016; Beumer and Clevers, 2021). Activated tuft cells secrete a diverse range of effector molecules, encompassing prostaglandin E2 and D2, cysteinyl leukotriene C4, acetylcholine, thymic stromal lymphopoietin, and β-endorphins. Notably, some of these molecules exhibit immunomodulatory properties. Tuft cells are critical for immune responses against helminthic and protozoan infections (Hendel et al., 2022).

Paneth cells are exclusively localized to the base of crypts of Lieberkuhn and release secretory granules filled with microbicidal proteins, including α-defensins and lysozyme, to defend against microbes (Ayabe et al., 2000). Paneth cells are strategically positioned near multipotent stem cells, where they regulate small intestinal epithelial cell renewal by secreting essential factors like EGF, WNT3, and Notch ligand DLL4 to support epithelial stem cell proliferation and epithelial renewal (Sato et al., 2011; Clevers and Bevins, 2013; Hooper, 2015). ISCs are thought to be interspersed between Paneth cells at the crypt base (identified by markers like LGR5) or at/near position 4 within the intestinal crypt (potentially expressing markers like DCAMKL-1 or BMI1) (Umar, 2010).

Goblet cells synthesize and secrete mucus to form a protective gel-like layer over the surface epithelium and defend it against bacterial invasion (Johansson et al., 2008). Goblet cells are responsible for synthesis and secretion of MUC2, the principal structural component of intestinal mucus (Pelaseyed et al., 2014). Unlike the gel-like mucus secreted by goblet cells, MUC1 is a transmembrane glycoprotein expressed on the apical surface of epithelial cells (Brayman et al., 2004). While small amounts of MUC1 are typically present in the normal intestine, its abundance is notably higher in the stomach (Lindén et al., 2009; Pelaseyed et al., 2014). It serves a crucial role in protecting these cells by preventing bacterial adhesion and inhibiting apoptosis (Lindén et al., 2009).

Goblet cells also secrete hydrophilic glycoproteins, including protective factors such as Anterior gradient 2 (AGR2), Zymogen granule protein 16 (ZG16), Trefoil Factor 3 (TFF3), Fc fragment of IgG binding protein (FCGBP), and Resistin-like Molecule β (RELM β). These secreted molecules contribute to a lubricative barrier, which blocks microbial invasion into the intestinal epithelium (Mashimo et al., 1996; Farrell et al., 2002; Huang et al., 2021). Goblet cells can actively acquire soluble antigens from the intestinal lumen and transport them to subepithelial dendritic cells, revealing a multifaceted role in immune interactions (Mcdole et al., 2012; Hooper, 2015).

Approximately one percent of gut epithelium is composed of rare and intricate enteroendocrine cells, constituting at least eight subsets characterized by the hormones they synthesize, such as enterochromaffin cells (e.g., serotonin, 5-HT), D cells (e.g., somatostatin), and G cells (e.g., gastrin) (Rezzani et al., 2022). The gastrointestinal tract is responsible for about 95% of the body’s 5-HT synthesis. Notably, enterochromaffin cells dynamically release stored 5-HT from intracellular secretory granules in response to various stimuli, including environmental factors, gut microbiota, mechanical stimulation, and metabolites. 5-HT stimulates visceral sensation, influences intestinal motility, and influences intestinal permeability (Chen et al., 2021b; Rezzani et al., 2022).

Microfold cells, commonly referred to as M cells, are specialized intestinal epithelial cells with a primary role in antigen sampling. These cells are predominantly located in the follicle-associated epithelium overlaying the surfaces of intestinal lymphoid tissues, including Peyer’s patches and isolated lymphoid follicles. M cells play a crucial role in antigen presentation within the intestinal tract (Kraehenbuhl and Neutra, 2000; Hooper, 2015).

#### Airway epithelium: organization and function

The human respiratory system is divided into the proximal conducting airway, encompassing the nasal cavity, trachea, and bronchi, and the distal respiratory airway, which includes the respiratory bronchioles and alveoli (Patton and Byron, 2007; Barkauskas et al., 2017; Davis and Wypych, 2021). A dynamic shift occurs in the proportion and biological characteristics of respiratory epithelial cells along the proximal-distal axis. In the small airway epithelium, which includes distal airways, a notable absence of mucus-producing cells is observed. Instead, secretoglobin-producing (“club”) cells occupy this region. The abundance of basal cells gradually diminishes with each subsequent airway branch, but basal cells persist throughout the human tracheobronchial tree, differing from mice, where they are absent in small airways (Mercer et al., 1994; Patton and Byron, 2007; Rock et al., 2010; Swangchan-Uthai et al., 2013; Yang et al., 2017).

The large airways, from the nose to the terminal bronchioles consists of pseudostratified columnar epithelium. This epithelium is integral to respiratory function, including mucus movement and airway protection (Patton and Byron, 2007; Crystal et al., 2008).

The transition from pseudostratified columnar epithelium in larger airways to cuboidal epithelium in terminal bronchioles is marked by characteristics such as secretory club cells, fewer multiciliated cells, and infrequent airway basal cells. The cuboidal epithelium possibly optimizes the balance between protective mechanisms and efficient gas exchange. As the terminal airways progress into alveoli, characterized by squamous alveolar cells, the emphasis shifts towards efficient gas exchange rather than active mucus clearance (Ali, 1965; Knudsen and Ochs, 2018).

**Figure 3:**
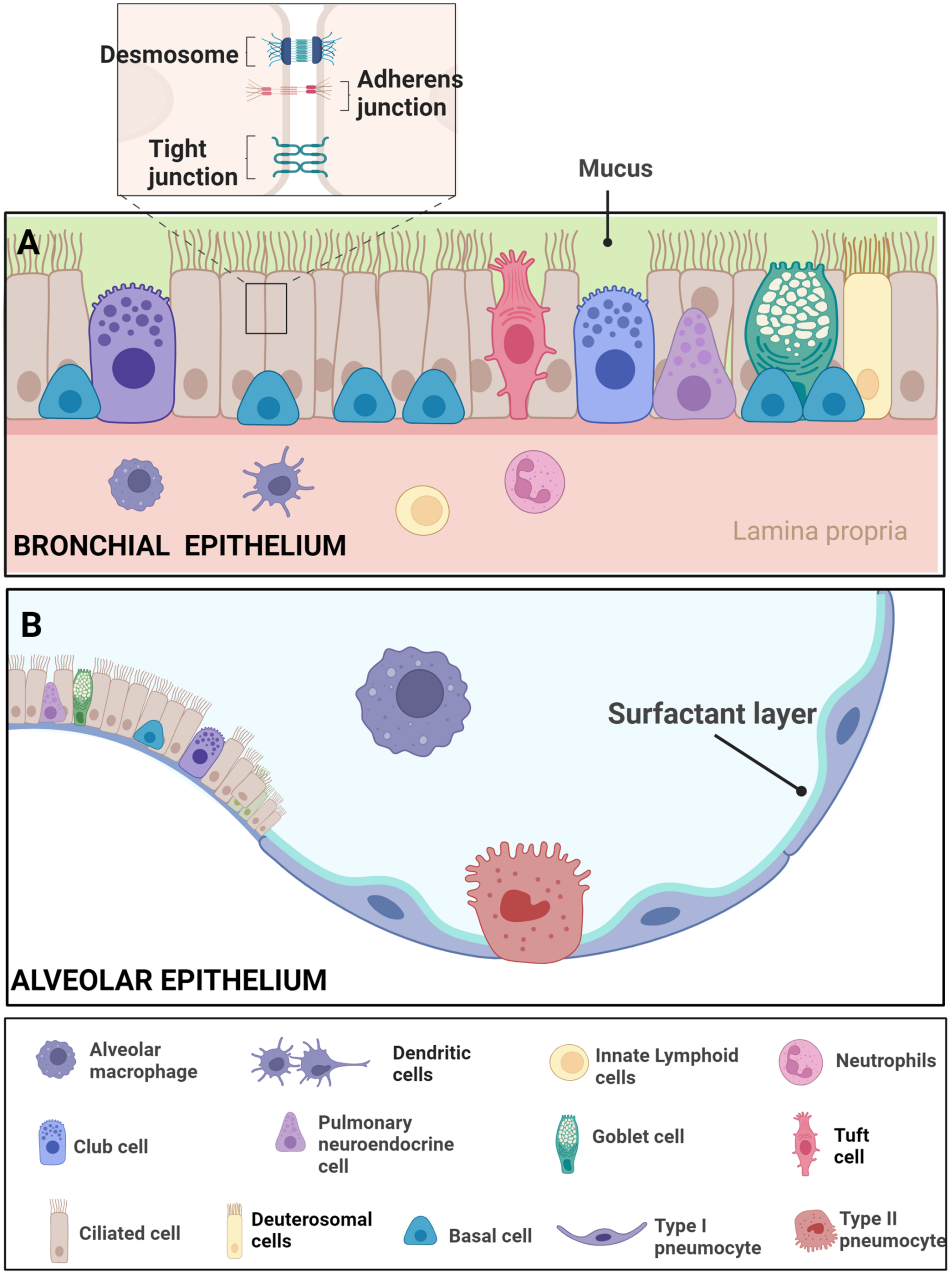
Epithelial features of the airway- Respiratory Epithelial Dynamics: **(A)** The bronchial epithelium orchestrates respiratory function and integrity through a diverse array of specialized cell types, intricately connected by junctional complexes. Basal cells, serving as primary stem cells, contribute to post-injury epithelial regeneration, while intermediate progenitor cells maintain airway tissue homeostasis. Club cells, involved in repair, exhibit the ability to dedifferentiate into basal cells. Ciliated cells, pervasive throughout the airways, coordinate mucociliary clearance through rhythmic ciliary movement. Goblet cells, vital for clearance, produce mucus containing diverse components such as mucins. Pulmonary neuroendocrine cells (PNECs) act as communication intermediaries, releasing neurotransmitters like 5-HT to bridge the immune and nervous systems. Tight junctions, adherens junctions, and desmosomes intricately connect these cell types, ensuring epithelial integrity amidst the dynamic airway environment. The figure illustrates resident macrophages, dendritic cells, innate lymphoids, and neutrophils. **(B)** The alveolar epithelium comprises Alveolar Type I cells, specialized for gas exchange, and Alveolar Type II cells, responsible for surfactant production to maintain alveolar integrity. Resident macrophages in the alveoli contribute to immune defense Adapted from “Respiratory epithelium”, by BioRender.com (BioRender, 2023).

The respiratory system’s capacity for repair, regeneration, and remodeling hinges on the functionality of adult progenitor cells. Normally, the turnover rate of lung cells is relatively low compared to highly regenerative tissues like the intestine (Rawlins, 2008; Schneider et al., 2021). Efficient airway regeneration mechanisms have been extensively studied in mice, establishing basal cells as the primary airway stem cells. Basal cells stand out as multipotent stem cells in the surface airway epithelium of conducting airways. Positioned along the airway basal lamina, basal cells establish connections with various luminal cell types, playing a pivotal role in homeostasis by possessing the ability to self-renew and differentiate into diverse luminal cell lineages. Basal cells give rise to various cell types, including ciliated cells and secretory cells such as goblet cells, club cells, ciliated cells, tuft cells, pulmonary neuroendocrine cells (PNECs), deuterosomal cells, and pulmonary ionocytes (**Figure 3**). Studies on tracheal epithelial cells in culture identified a novel cluster termed “pulmonary ionocytes” expressing genes related to ion transport and pH (Hewitt and Lloyd, 2021).

Club cells (also known as Clara cells) are secretory cells predominantly found in the terminal and respiratory bronchioles. Positioned luminal to the epithelium, club cells establish connections with the lamina propria. The location of club cells enables them to engage with both the inner airway environment and the surrounding connective tissue. Club cells exhibit a columnar shape and contain secretory granules filled with anti-microbial and anti-inflammatory peptides. They contribute to xenobiotic metabolism, with SCGB1A1 serving as a well-studied marker. Lineage tracing studies indicate that club cells give rise to multiciliated cells, identified by the expression of FOXJ1 (transcription factor necessary for cilia formation), and goblet cells, expressing mucin MUC5AC (Zaragosi et al., 2020). In certain scenarios, club cells may contribute to the basal stem cell pool, although the physiological implications of this response in injury remain unclear (Tata et al., 2018; Basil et al., 2020).

Ciliated cells, crucial for moving the mucus blanket, exhibit transcriptionally distinct subsets along the proximal-distal axis (Travaglini et al., 2019, 2020). Travaglini et al. utilized C20orf85 as a general ciliated marker and DHRS9 as a proximal ciliated marker to label and quantify different types of ciliated cells in various regions of the airway epithelium through single-molecule fluorescence *in situ* hybridization. However, the functional roles of distinct ciliated cell types identified by these markers remain to be elucidated (Travaglini et al., 2019, 2020). Ciliated cells play a pivotal role in mucociliary clearance (MCC), trapping and expelling microorganisms, mucus, and debris through the rhythmic beating of cilia (Bustamante-Marin and Ostrowski, 2017; Davis and Wypych, 2021). Deuterosomal cells mark an intermediate cell state before the differentiation of multiciliated cells and play a key role during MCC differentiation. Though fewer in number than the MCC population, each deuterosomal cell transits quickly through this stage (Ruiz García et al., 2019).

Goblet cells, identified by their goblet-like appearance, are the primary mucus-producing cells in the airways. Partnering with ciliated cells, they contribute to effective MCC, producing mucus containing various components such as electrolytes, metabolites, fluids, antimicrobial products, and mucins like MUC5AC and MUC5B (Ruysseveldt et al., 2021). While MUC1 is not typically produced by goblet cells, its significance in airway epithelial cells is increasingly recognized (Kato et al., 2017). Recent evidence suggests that MUC1 plays a central, anti-inflammatory role following the activation of host inflammation in response to a variety of infectious insults, such as *Pseudomonas aeruginosa* (Kim and Lillehoj, 2008; Dhar et al., 2017; Kato et al., 2017; Mcauley et al., 2017).

The respiratory tract is home to chemosensory epithelial cells, prominently featuring tuft cells. These specialized cells, distinguished by their tufted appearance, play a crucial role in sensing various environmental stimuli. Tuft cells are involved in orchestrating signaling pathways related to immune responses and maintaining tissue homeostasis within the respiratory system. Tuft cells, resembling taste cells, evoke responses from immune and neuronal cells. While their role in the lung is less certain, tuft cells have been detected in the nose, trachea, and proximal airways, mediating communication between neuronal and immune pathways (Hewitt and Lloyd, 2021).

PNECs, found as solitary cells or within clusters (neuroendocrine bodies - NEBs), act as crucial intermediaries between the immune and nervous systems. These rare, epithelial-resident cells sense airway activity and produce 5-HT and other peptides to stimulate immune responses. Despite constituting a small fraction of all epithelial cells in the human airway, the normal function and role of PNECs in lung disease is only beginning to be understood (Davis and Wypych, 2021; Hewitt and Lloyd, 2021).

The transition from terminal airways into alveoli involves a shift in cellular composition, featuring squamous alveolar type 1 (AT1) cells and cuboidal alveolar type 2 (AT2) cells. This cellular transformation occurs in the transition between proximal to distal airways. AT1 are large squamous cells that cover 95% of the alveolar surface, constituting the primary epithelial component of the air–blood barrier. These cells play a crucial role as they constitute the major gas exchange surface of the alveolus, contributing significantly to the maintenance of the permeability barrier function of the alveolar membrane. On the other hand, AT2 pneumocytes serve as the progenitors of type 1 cells and are responsible for the production of surfactant, essential for maintaining proper lung function and homeostasis. The intricate cellular arrangement in the distal airway underscores the specialization of cell types in the alveoli to support efficient gas exchange and respiratory well-being (Haies et al., 1981; Castranova et al., 1988; Stone et al., 1992).

### Pathogen detection

The epithelium plays a crucial role in microbial detection under normal and pathological conditions. Evolutionarily conserved Pattern Recognition Receptors (PRRs) are key to host survival, as emphasized by Larsen et al. (2020) (Larsen et al., 2020). PRRs, which are found on various cell types including intestinal epithelial cells (IECs) and airway epithelial cells, as well as immune cells, are essential for detecting microbial presence and products within their respective environments. This capability places IECs and airway epithelial cells at the junction of a bidirectional interaction between mucosal immune cells and the external environment (Li and Wu, 2021).

Major classes of PRRs include transmembrane proteins like Toll-like receptors (TLRs) and C-type lectin receptors (CLRs), as well as cytoplasmic proteins such as Retinoic acid-inducible gene (RIG)-I-like receptors (RLRs) and NOD-like receptors (NLRs). Upon activation, PRRs initiate downstream signaling cascades, inducing innate immune and inflammatory responses. The signaling cascades cause immune cells, including macrophages, dendritic cells (DCs), neutrophils, and other nonprofessional immune cells to synthesize proinflammatory cytokines, type I interferons (IFNs), major histocompatibility (MHC) proteins, and costimulatory molecules. TLRs and NLRs, as two major PRR subfamilies, provide immediate responses against pathogenic invasion or tissue injury, recognizing pathogen-associated molecular patterns (PAMPs) from microbes or self-molecules (Naik et al., 2018; Wicherska-Pawłowska et al., 2021).

Epithelial cells in tissues like the intestine and airways respond to PAMPs or danger-associated molecular patterns (DAMPs) by activating specific signaling pathways via their respective PRRs. Additionally, antigen-presenting cells like dendritic cells, macrophages, and B cells are stimulated by PAMPs and DAMPs from stressed or damaged tissues or microbes (Rakoff-Nahoum et al., 2004; Lavelle et al., 2010).

During microbial infections, PAMPs originating from various organisms but absent in the host act as external signals to alert the immune system to the presence of pathogens, prompting immune responses. Conversely, DAMPs released by cells serve as endogenous signals, indicating unscheduled cell death, microbial invasion, or stress (Bianchi, 2007; Herwald and Egesten, 2016). Additionally, PRRs, together with host factors, may contribute to the pathogenicity and diverse manifestations of diseases (Mogensen et al., 2006; Mogensen, 2009). These interactions highlight the intricate balance between microbial detection and disease progression.

PAMPs possess highly conserved structures essential for pathogen survival, integrity, and function. This conservation allows the immune system to broadly recognize and respond to a diverse range of pathogens without prior exposure. Major PAMPs encompass microbial nucleic acids (e.g., unmethylated CpG motifs, dsRNA, ssRNA), lipoproteins, surface glycoproteins, and membrane components (such as peptidoglycans, lipopolysaccharide, and glycosylphosphatidylinositol) (Tang et al., 2012). PRRs detect the PAMPs, enabling the immune system to discern ‘self’ from ‘non-self’ and trigger innate immune signals. DAMPs, arising from trauma, ischemia, or non-pathogenic tissue damage, can initiate and sustain immune responses. DAMPs manifest within various cellular compartments, including the nucleus (HMGB1), cytoplasm (S100 proteins), exosomes (heat shock proteins), the extracellular matrix (e.g., hyaluronic acid), and plasma components like complement proteins (C3a, C4a, C5a) (Bianchi, 2007; Tang et al., 2012; Herwald and Egesten, 2016). Examples of non-protein DAMPs comprise ATP, uric acid, heparin sulfate, RNA, and DNA, and they play significant roles in inflammatory diseases like sepsis (Tang et al., 2012).

**Figure 4:**
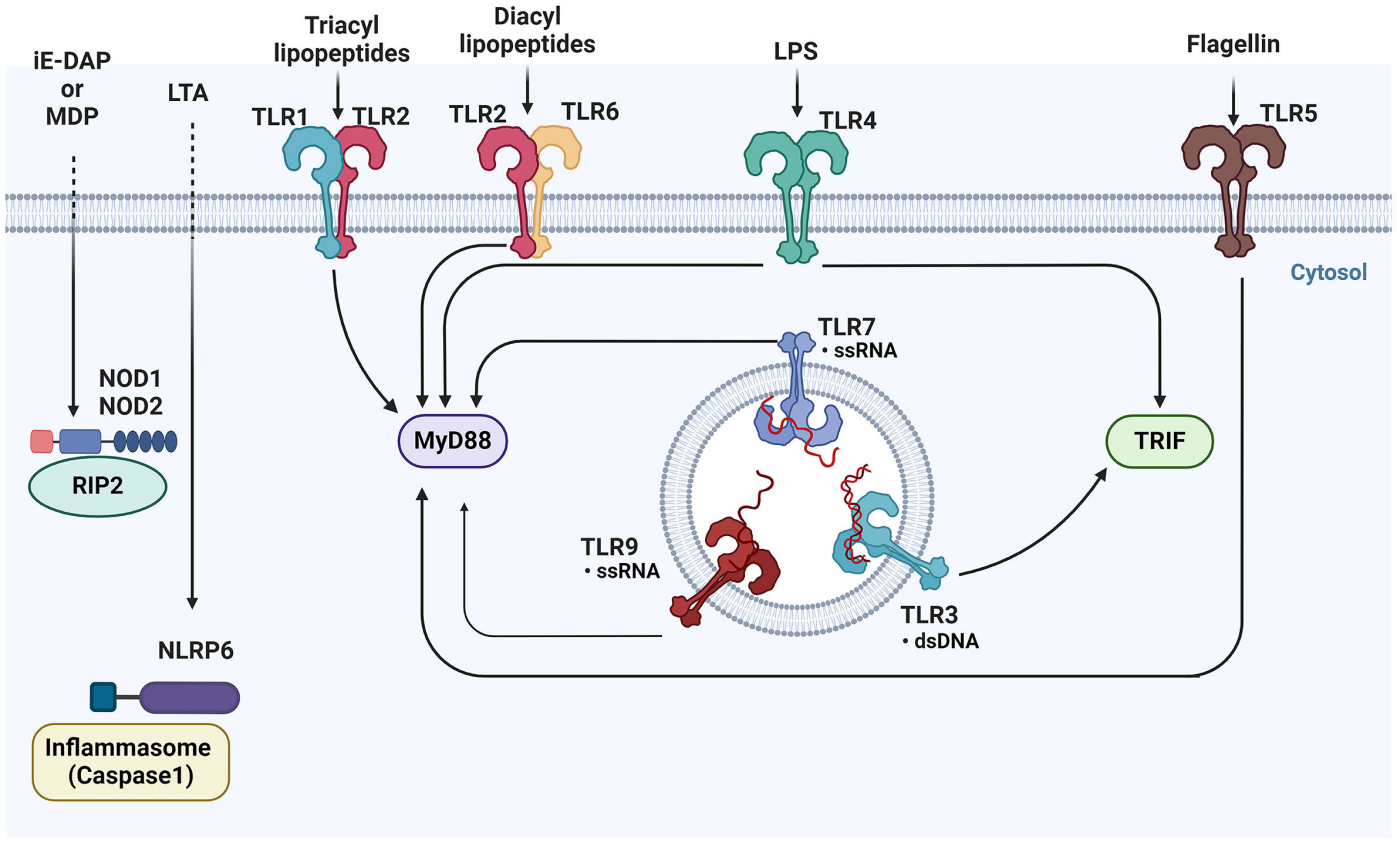
Overview of pattern recognition receptors and their ligands in epithelial cells- This figure illustrates the diverse array of Pattern Recognition Receptors (PRRs) and their corresponding ligands. TLRs, located on the cell surface and in endosomes, recognize a variety of microbial components, including lipids, proteins, and nucleic acids. Upon activation, TLRs signal through adaptor proteins such as MyD88 and TRIF to initiate downstream immune responses. Meanwhile, cytoplasmic NOD1 and NOD2, detect bacterial peptidoglycans in the cytosol. TLRs, Toll-like receptors; MyD88, Myeloid differentiation primary response 88; TRIF, Toll/interleukin-1 receptor domain-containing adapter-inducing interferon-β; NOD1, Nucleotide-binding oligomerization domain-containing protein 1; NOD2, Nucleotide-binding oligomerization domain-containing protein 2 (BioRender, 2023).

TLRs and C-type lectin receptors (CLRs) are crucial for recognizing fungal cell wall components (Jannuzzi et al., 2020). Viral infections are detected by various PRRs, including TLRs, retinoic acid-inducible gene I-like receptors (RLRs), and cytosolic DNA sensors. These receptors recognize viral nucleic acids and trigger the production of interferons and cytokines to combat viral infections (Thompson et al., 2011). Bacterial infections, on the other hand, activate PRRs like TLR5, TLR4, TLR2, TLR9, NOD1, and NOD2, which recognize bacterial cell wall components or nucleic acids (**Figure 4**) (Akira et al., 2006; Mogensen, 2009). These receptors are crucial for initiating immune responses against bacterial pathogens (more details **Table 2**) (Akira et al., 2006; Hirata et al., 2007; Ireton and Gale, 2011; Thompson et al., 2011; Jannuzzi et al., 2020).

**Table 2:** Microbial components, species, and corresponding pattern recognition receptors.

Microbial Components	Species	PRRs
Bacteria		
Triacyl lipopeptide	Bacteria and mycobacteria	TLR1/TLR2
PG	Gram-positive bacteria	TLR2
Porins	*Neisseria*	TLR2
Lipoarabinomannan	Mycobacteria	TLR2
Diacyl lipopeptides	*Mycoplasma*	TLR6/TLR2
LTA	Group B *Streptococcus*	TLR6/TLR2
LPS	Gram-negative bacteria	TLR4
Flagellin	Flagellated bacteria	TLR5
CpG-DNA	Bacteria and mycobacteria	TLR9
Diaminopimelic acid	Gram-negative bacteria	NOD1
MDP	Gram-positive and -negative bacteria	NOD2
Fungus		
Phospholipomannan	*Candida albicans*	TLR2
Mannan	*Candida albicans*	TLR4
Glucuronoxylomannan	*Cryptococcus neoformans*	TLR2 and TLR4
Zymosan	*Saccharomyces cerevisiae*	TLR6/TLR2
β-glucan	Candida albicans, Aspergillus fumigatus	Dectin-1
Alfa-mannan	Candida albicans, Aspergillus fumigatus	Dectin-2
Viruses		
Hemagglutinin protein	Measles virus	TLR2
dsRNA	Viruses	TLR3
Envelope proteins	RSV, MMTV	TLR4
DNA	Viruses	TLR9
dsRNA (short), 5-triphosphate RNA	Viruses	RIG-1
dsRNA (long)	Viruses	MDA5

Beyond PRRs, intracellular pathways linking immune and inflammatory responses to ion channels have been identified. Transient receptor potential (TRP) channels, a major family of non-selective cation permeable channels, play a significant role as cellular sensors. TRP channels have been implicated in the pathogenesis of numerous inflammatory diseases (Gees et al., 2010; Han and Yi, 2014). Next, we will specifically highlight the contributions of TLRs, NLRs, and TRP channels in microbial detection and the orchestration of immune and inflammatory responses.

#### Toll-like receptors

The first receptors encoded by the mutated toll gene were described in fruit flies (*Drosophila melanogaster*) (Nüsslein-Volhard and Wieschaus, 1980). TLRs, innate molecules distributed on both epithelial and immune cells, serve as key sentinels by recognizing conserved molecular motifs on bacteria and viruses. TLRs exhibit a tripartite domain architecture characterized by an extracellular ligand binding domain (ECD) comprising leucine-rich repeats (LRR), a singular transmembrane (TM) domain, and an intracellular Toll/interleukin-1 receptor (TIR) domain (ICD) (Botos et al., 2011). Activation of TLRs by ligands initiates intracellular signaling cascades, leading to the production of cytokines, chemokines, and the transcription of genes crucial for infection control. Upon ligand binding, TLRs form dimers, facilitating the association of intracellular TIR domains and recruitment of adaptor proteins like MYD88 and TRIF (Kenny et al., 2009; Fitzgerald and Kagan, 2020).

MYD88, a crucial adaptor molecule, contributes significantly to TLR signaling (excluding TLR3) and non-TLR pathways, including IL-1R signaling (Kenny et al., 2009). TLRs initiate immune responses in the epithelium, predominantly activating MAP kinases and key transcription factors, including Nuclear factor kappa B (NF-κB), Interferon regulatory factor 3 (IRF-3), and interferon regulatory factor 7 (IRF-7), resulting in the induction of proinflammatory cytokines and type I and type III interferons (Vareille et al., 2011). TLRs are distributed across the cell and their subcellular distribution influences their roles in the immune response. TLR2 is positioned sub-apically on the plasma membrane (P.M.), while TLR2/1 and TLR2/6 are basolateral. TLR3 resides in endosomes and on both the luminal and basal sides of the plasma membrane. TLR4 and TLR5 are basolateral on the P.M., and TLR9 is found in endosomes and at the basolateral side of the P.M. This spatial distribution is integral to their functionality in pathogen detection and immune activation.

The digestive system mucosa, closely connected to the oral and respiratory tracts, houses numerous commensal microorganisms. TLRs in the esophagus (TLR4, TLR2, and TLR3) respond to bacterial and viral components. In the small and large intestines, TLR expression is tightly regulated and TLR4, TLR2, TLR5, and TLR9 are localized to specific regions. TLR activation in intestinal epithelial cells leads to cytokine secretion and involves M cells, Paneth cells, and goblet cells, playing crucial roles in antigen sampling, immune responses, and antimicrobial substance production, controlling pathogens and commensals (Kamdar et al., 2013; Allaire et al., 2018) (More information can be found in **Table 3**). TLRs regulates crypt dynamics by influencing the proliferation and apoptosis of stem cells and transit amplifying cells. TLRs recognize microbial motifs, contributing to the enhancement of the intestinal epithelial barrier function, tightening intercellular junctions, promoting the secretion of mucus and antimicrobial peptides, and generating reactive oxygen species (Burgueño and Abreu, 2020).

**Table 3:** PRRs in the epithelium (airway/gut).

Receptors	Cellular Location	Cell type	Ligand	Adaptor protein	Reference
Toll-like receptors					
TLR2/1	P.M. (Basolateral)	Goblet cells, Enterocytes, EECs, Club cells, AT2, Ciliated cells	Triacylated lypopeptides	MYD88	(Mcclure and Massari, 2014; Price et al., 2018; Sampaio et al., 2018; Atlas, 2024)
TLR2	P.M. (Sub-apical)	Goblet cells, Enterocytes Paneth cells, Club cells, AT1, AT2, Ciliated cells	Biglycan, Decorin, Versican, LMW hyaluronan, S100 proteins, Heat shock proteins, Aβ, Histones, HMGB1, EDN	MYD88	(Price et al., 2018; Roh and Sohn, 2018; Atlas, 2024)
TLR2/6	P.M. (Basolateral)	Goblet cells, Enterocytes, EECs, Club cells, AT1, AT2, Ciliated cells	Diacylated lipopeptides	MyD88	(Li et al., 2013; Price et al., 2018, Atlas, 2024)
TLR3	Endosome, P.M. (Luminal and Basal)	Goblet cells, Enterocytes, Paneth cells, EECs, Club cells, AT1, AT2, Ciliated cells	dsDNA, RNA	TRIF	(Price et al., 2018; Roh and Sohn, 2018; Chen et al., 2021a; Atlas, 2024)
TLR4	P.M. (Basolateral)	Goblet cells, Enterocytes, EECs, Club cells, AT1, AT2, Ciliated cells	Biglycan, Decorin, LMW hyaluronan, Heparan sulfate, Fibronectin, Fibrinogen, Tenascin C, S100 proteins, Heat shock proteins, Histones, HMGB1, HMGN1, Defensins, Granulysin, Syndecans, Glypicans,LPS, LBP	MYD88, TRIF	(Price et al., 2018; Roh and Sohn, 2018; Ciesielska et al., 2021; Atlas, 2024)
TLR5	P.M. (Basolateral)	Paneth cells, Goblet cells, Enterocytes, EECs (colon), Club cells, AT1, AT2, Ciliated cells	Flagellin	MyD88	(Price et al., 2018; Hussain et al., 2020; Atlas, 2024)
TLR9	Endosome, P.M. (Basolateral)	Enterocytes, AT1, AT2	CpG ODN Diacylated lipopeptides, ODNs, NTHI, DNA, mtDNA	MYD88	(Sanjuan et al., 2006; Price et al., 2018; Roh and Sohn, 2018; Atlas, 2024)
NOD-like receptors					
NOD1	Cytosol	Enterocytes, Goblet cells, Paneth cells, EECs (only colon) Club cells, AT1, AT2, Ciliated cells	D-glutamyl-meso-diaminopimelic acid	RIP2, ATGL16	(Leiva-Juárez et al., 2018; Atlas, 2024)
NOD2	Cytosol	Enterocytes, Paneth cells, EECs, Club cells, AT2, Ciliated cells	MDP	RIP2, ATGL16	(Sidiq et al., 2016; Atlas, 2024)
NLPR6	Cytosol	Enterocytes, Goblet cells	LPS, ATP	ASC	(Venuprasad and Theiss, 2021; Atlas, 2024)
RIG-I-like receptors					
RIG-1	Cytosol	Enterocytes, Goblet cells, Paneth cells, AT1, AT2, Ciliated cells, Club cells	IPS-1	dsRNA (short), 5-triphosphate RNA	(Loo and Gale, 2011)
MDA5	Cytosol	Enterocytes, Goblet cells, Paneth cells, EECs, AT1, AT2, Ciliated cells, Club cells	IPS-1	dsRNA (long)	(Loo and Gale, 2011)
C-type lectin receptors					
Dectin-1	P.M.	IEC, AT1, AT2, Ciliated cells, club cells	Beta-glucan	TK-dependent and non-TK dependent pathways	(Cohen-Kedar et al., 2023)
Dectin-2	P.M.	IEC, Respiratory epithelium	Alfa-mannan	TK-dependent and non-TK dependent pathways	(Cohen-Kedar et al., 2023)

LBP, lipopolysaccaride-binding protein; LPS, lipopolysaccharide; MDP, muramyl dipeptide; NTHi, non-typeable Haemophilus influenza; ODN, oligodeoxynucleotide; P.M., plasma membrane; EDN, eosinophil-derived neurotoxin; EECs, enteroendocrine cells; AT1, alveolar type 1; AT2, alveolar type 2; ASC, Apoptosis-associated speck-like protein containing a CARD; TK, Tyrosine Kinase.

TLR expression in airway epithelium is pivotal for immune responses and varies based on physiological or disease states. TLR2 and TLR4 are essential and become more abundant during infections and inflammation. TLR3 and TLR5 also contribute to airway defense against viral and bacterial components (Parker and Prince, 2011; Mcclure and Massari, 2014). TLR activation in the upper respiratory epithelium leads to mucus and antimicrobial protein production, while the lower respiratory tract primarily relies on TLR4 signaling, producing substances like defensins, lysozyme, nitric oxide, and cytokines. TLR activation prompts recruitment of neutrophils, eosinophils, monocytes, and dendritic cells (Mcclure and Massari, 2014).

#### NOD-like receptors (NLRs)

NLRs, a highly conserved group of cytosolic receptors, play a vital role in recognizing DAMPs and PAMPs. Their common structure consists of a central Nucleotide-binding and Oligomerization Domain (NOD) and a Leucine-Rich Repeat (LRR) in the C-terminal. The NLR family is divided into two subfamilies based on their N-terminal domain: NLRC, featuring caspase-activating and recruiting domains (CARDs), and NLRP, comprising proteins with a pyrin domain (Wicherska-Pawłowska et al., 2021; Maruta et al., 2022).

The NLRC subfamily includes NOD1, NOD2, NLRC4, NLRX1, NLRC3, and NLRC5, while the NLRP subfamily consists of 14 NLRPs with a pyrin domain. Activation of NLRs by DAMPs or PAMPs leads to NF-κB and MAP kinase signaling cascade activation or inflammasome formation (Davis et al., 2011).

NLRP6, abundantly expressed in the intestinal epithelium, plays a pivotal role in maintaining intestinal health and microbiota balance. In the intestinal landscape, NLRP6 regulates microbiome composition, preserving homeostasis. Co-modulated by microbiota-derived metabolites, NLRP6 governs epithelial IL-18 secretion and antiviral responses. Goblet cells, crucial for mucosal defense, are influenced by NLRP6, challenging traditional perspectives (Wlodarska et al., 2014; Zheng et al., 2020a). Beyond the gut, studies unveil a novel role for NLRP6 in governing lung inflammation induced by cigarette smoke, linking the gut-lung axis. During lung infections, NLRP6 exhibits diverse effects depending on the microbe (Ghimire et al., 2018; Xu et al., 2021; Shi et al., 2022). NLRP6 plays a damaging role in the intestinal phase of Brucella infection (Rungue et al., 2021). Additionally, considering NLRP6’s antiviral role in the intestines, it would be interesting to investigate if NLRP6 plays a protective or negative role in pulmonary host defense during viral infection (Ghimire et al., 2018).

In the intestine, NLRs contribute to mucosal integrity and homeostasis by regulating tolerance to commensal microbiota and controlling inflammatory signaling. Experimental and clinical evidence supports the protective role of NLR-mediated signals in intestinal inflammation (Lavelle et al., 2010). NOD1 and NOD2 distinguish between Gram-negative and Gram-positive bacteria by sensing specific peptidoglycan motifs. IECs and small intestinal Paneth cells express NOD2, observed at higher levels in Paneth cells of inflammatory bowel disease (IBD) patients. Inflammation in IBD contributes to increased NOD2 signaling (Claes et al., 2015). Upon ligand sensing, NOD1 and NOD2 recruit the adaptor kinase receptor-interacting protein 2 (RIP2), leading to the production of antimicrobial peptides and pro-inflammatory cytokines (Kufer et al., 2006; Franchi et al., 2009; Claes et al., 2015). More information about NOD1 and NOD2 can be found in **Table 3**.

Lung epithelial cells express NLRs, particularly NOD1 and NOD2, which recognize bacterial peptidoglycan components. Deficiencies or polymorphisms in these receptors can increase susceptibility to respiratory infections, and NOD1 variants are linked to asthma (Trindade and Chen, 2020). NLRP1 enhances resistance against pneumonia, NLRP3 detects DAMPs in bronchial epithelial cells during infection, and NLRC4 triggers inflammasomes in response to specific bacteria. NLRX1, with a mitochondrial targeting sequence, contributes to ROS production (Leiva-Juárez et al., 2018).

#### Transient receptor potential

TRP channels, a family of non-selective cation-permeable channels, serve as versatile cellular sensors involved in various physiological processes. The 28 identified mammalian TRP channels are classified into six subfamilies: TRPC, TRPV, TRPM, TRPP, TRPML, and TRPA (Samanta et al., 2018).

Among TRP channels, transient receptor potential ankyrin (TRPA) channels, specifically TRPA1, are Ca^2+^ permeable nonselective cation channels conserved throughout the animal kingdom. TRPA1 possesses 14 ankyrin repeats in its NH2-terminus, a unique structural feature relevant to its interactions with intracellular components. TRPA1 plays a crucial role in detecting a variety of exogenous stimuli, contributing to cellular damage responses. TRPA1 is expressed not only in sensory neurons but also in various non-neuronal cell types, including lung and intestine epithelial cells, impacting acute and chronic pain and inflammation across organ systems (Nassini et al., 2015; Tian et al., 2015; Talavera et al., 2020).

Epithelial TRPA1 receptors are vigilant sensors, detecting environmental irritants and potential threats. When activated, TRPA1 sets off signaling pathways that signal the body to impending dangers, initiating defensive measures such as the release of inflammatory mediators and neuropeptides. Additionally, TRPA1 regulates epithelial barrier integrity, modulates secretion, and coordinates protective responses to preserve the function of the epithelial barrier and safeguard against potential hazards (Brierley et al., 2009; Luostarinen et al., 2023).

In the intestine, TRPA1 activation by microbes was not well understood. Chen et al. (2021b) demonstrated that in the gut, IL-33 transduces a non-canonical signaling pathway, inducing robust Ca^2+^ influx in EECs, leading to 5-HT secretion. IL-33-mediated 5-HT release in EECs is TRPA1-dependent, uncovering a gut sensation machinery that regulates intestinal homeostasis and host defense against enteric infection (Chen et al., 2021b). TRPA1+ EECs mediate 5-HT release, enhancing intestinal motility critical for pathogen expulsion and contributing to the host’s defense against enteric infections. Moreover, Ye et al. (2021) discovered that the bacterium *Edwardsiella tarda* activates enteroendocrine cells through the TRPA1 receptor, promoting intestinal motility crucial for clearing parasites and maintaining gut health. This suggests that TRPA1+ enteroendocrine cells may serve as a host protective mechanism (Vallance and Collins, 1998; Ye et al., 2021).

Contrary to the initial belief that TLR4 was the sole pattern recognition receptor for LPS, emerging evidence suggests that TRPA1 (Ca^2+^permeable channel) also acts as a membrane-bound sensor of LPS (Boonen et al., 2018; Ko et al., 2020; Mazgaeen and Gurung, 2020). LPS, a constituent of the outer membrane of Gram-negative bacteria, activates TRPA1 in human bronchial epithelial cells, leading to increased Ca^2+^ influx. This rise in intracellular Ca^2+^ triggers NADPH oxidase activation, elevating intracellular ROS levels. The increased ROS, in turn, activates the MAPK/NF-κB signaling pathway, resulting in IL-8 induction. Notably, TRPA1 appears to sense LPS in a manner that is independent of TLR4 (Ko et al., 2020). These findings provide valuable insights into the pathogenic mechanisms associated with TRPA1-mediated, LPS-induced lung inflammation and may contribute to the development of potential therapies (Ko et al., 2020).

### Antimicrobial effector molecules

Antimicrobial Proteins and Peptides (AMPs) defend against microbial threats, contributing significantly to the maintenance of mucosal integrity and host-flora homeostasis (Zhang et al., 2021). Epithelial cells in the gut, skin, and respiratory tract deploy a diverse array of AMPs, reflecting the intricate microbial challenges faced by these tissues. Under normal circumstances, epithelial cells act as the primary source of AMPs in body surface tissues. However, during inflammation, infiltrating immune cells also produce AMP (Gallo and Hooper, 2012).

In baseline conditions, epithelial cells constitutively produce and release AMPs, such as cathelicidins and defensins, as part of their fundamental defense against various pathogens, including bacteria, fungi, and viruses. These peptides exhibit broad-spectrum antimicrobial activity by disrupting microbial cell membranes, thwarting pathogen colonization or invasion. Cathelicidins and defensins selectively target harmful microorganisms without disrupting commensal or beneficial bacteria (McDermott, 2004; Leonard et al., 2012).

Defensins influence immune responses by interacting with immune cells, such as dendritic cells and T cells, regulating inflammation and modulating adaptive immune responses. Cathelicidins and defensins also participate in wound healing and tissue repair processes by promoting cell proliferation and migration to restore tissue integrity after injury (Raj and Dentino, 2002; Tecle et al., 2010; Xu and Lu, 2020).

It’s noteworthy that cathelicidins often exhibit different molecular structure than defensins, conferring unique and non-overlapping antimicrobial mechanisms and immune-modulatory effects. The diverse actions of cathelicidins and defensins adds an extra layer of complexity to their functions (Agier et al., 2015).

Upon activation of PRRs, epithelial cells initiate cytokine signals for leukocyte-mediated responses and produce various effector molecules with direct microbicidal effects, offering potential therapeutic targets (Leiva-Juárez et al., 2018).

While AMPs are recognized as a first line of defense, their diversity and specificity in response to different pathogens makes it challenging to establish pathogen specificity (Wassing et al., 2015). The first AMP was discovered in 1981 by Hans G. Boman and colleagues and since then, the repertoire has expanded to include over 2300 naturally occurring AMPs (Nakatsuji and Gallo, 2012).

Human AMPs are categorized into families based on cationic (polar) structures, hydrophobic regions and charge separation, and serve as peptide antibiotics (Fearon and Locksley, 1996; Mahlapuu et al., 2016). AMPs exhibit variations across families in size, amino acid sequence, and structural motifs, with specific genes encoding each peptide. The organization and chromosomal location of AMPs across mammalian species provide insights into the evolutionary development of this host defense system (Chaplin, 2010). The defensins and cathelicidins expressed in human epithelial tissues are shown in **Table 4**. We further review the two most common AMPs in humans: cathelicidins and defensins.

**Table 4:** Antimicrobial peptides (AMPs) expressed in human epithelial tissues.

AMP family	Example	Epithelial tissues	Cellular functions	Reference
α-defensins	HD5, HD6	Paneth cells, Enterocytes, Goblet cells, EECs	Chemoattraction of immune cells, phagocytosis, cytokine induction, and anti-inflammatory properties	(Ouellette, 2011; Fruitwala et al., 2019; Atlas, 2024)
β-defensins	hBD-1/-2/-3	Enterocytes, Goblet cells, EECs, Paneth cells, Club cells, AT1, AT2, Ciliated cells	Chemoattraction of immune cells, cytokines chemokines induction, modulation of cellular functions and differentiation/activation markers	(Niyonsaba et al., 2004; Fruitwala et al., 2019; Atlas, 2024)
Cathelicidins	LL-37 (hCAP-18)	Enterocytes, EECs, Club cells, AT2, Ciliated cells	Chemoattraction of immune cells, activation of epithelial cells, epithelial wound repair, modulate cancer progression, and stimulate the secretion of chemokines	(Gordon et al., 2005; Atlas, 2024)

#### Defensins

Human defensins exist in the α and β forms, comprising a prominent AMP family with many homologous peptides found in human tissue.

α-defensins are divided into two major classes based on their expression patterns and gene structures: myeloid defensins or human neutrophil peptides (HNPs) 1 to 4 and human (enteric) defensins (HDs) 5 and 6. HNPs are stored in the azurophilic granules of human neutrophils, with HNPs 1–3 and their less abundant cousin HNP4 accounting for this storage. HD5 and HD6 are constitutively expressed in and secreted by Paneth cells at the bottom of the small intestinal crypt (Niyonsaba et al., 2004; Ouellette, 2011). Six human β-defensins (hBD-1 to -6) have been identified. While human β-defensin 1 (HBD1) is constitutively expressed, hBD2 and hBD3 are induced by microbial insults and pro-inflammatory cytokines in various epithelial and mucosal tissues (Xu and Lu, 2020).

Post-translational processing of β-defensins occurs similarly to cathelicidin and α-defensins (HD5 and HD6), with cleavage of the pro-peptide after secretion (Ouellette, 2011; Fu et al., 2023). The first human defensin discovered was hBD1, encoded by the DEFB1 gene. Constitutive expression by epithelial cells of the respiratory, intestinal, and urinary tracts and keratinocytes of the skin highlights the role of hBD1 in microbial infection protection (McDermott, 2004). hBD2 is mainly expressed by skin, respiratory, intestinal, and gingival epithelium, with variable levels in normal healthy cells and induction by various stimuli. hBD3 and hBD4 are less characterized but are found in the skin and respiratory epithelial cells, respectively, with inducible expression (Diamond et al., 2009; Nuding et al., 2009; Ouellette, 2011).

#### Cathelicidins

In humans, the exclusive cathelicidin is termed hCAP18/LL-37 (Lu and Stappenbeck, 2022). Immune cells, such as neutrophils, monocytes, lymphocytes, natural killer cells, and epithelial cells in the intestinal, respiratory, and urinary tracts are the main sources of cathelicidin in humans (Agier et al., 2015; Lu and Stappenbeck, 2022). Mechanisms that regulate cathelicidin expression vary by cell type and the makeup of inflammatory mediators or microbial structures such as LPS or lipoteichoic acid (LTA). Cathelicidin can be induced by compounds like the active form of vitamin D3, 1,25-dihydroxyvitamin D3 (VitD3) (Alford et al., 2020; Yang et al., 2020).

### Host-microbiota crosstalk

#### Epithelial interactions

The microbiota, with a specific emphasis on the gut and respiratory system within the context of this literature review, plays a pivotal role in sustaining host homeostasis and intricately regulating immune functions. While the gut microbiota has been extensively studied, investigation of airway microbiota is still evolving. Imbalances in the microbiota, known as dysbiosis, can lead to various diseases, including IBD, allergies and asthma, autoimmune diseases (such as rheumatoid arthritis). Understanding the complex interplay between microorganisms, the environment, including epithelial surfaces, and the host remains a key focus of ongoing research (Man et al., 2017).

In gut epithelium, a close and intricate relationship exists between epithelial cells and the microbiota. Gastrointestinal microbiota composition is influenced by diverse environmental factors, including pH, oxygen levels, nutrient availability, and temperature, fostering the thriving of various populations within the host’s environment (Milani et al., 2017).

The gut microbiota typically encompasses six phyla: *Firmicutes, Bacteroidetes, Actinobacteria, Proteobacteria, Fusobacteria*, and *Verrucomicrobia*. Among fungi, extensively studied species include *Candida, Saccharomyces, Malassezia*, and *Cladosporium* (Auchtung et al., 2018; Hou et al., 2022). The human gut microbiota also houses viruses, phages, and archaea (Lozupone et al., 2012; Wright et al., 2015; Hou et al., 2022). While extensive research has focused on bacterial components, the roles of fungi, viruses, and other microbes in health and disease remain inconclusive.

Contrary to the longstanding belief in the sterility of the lung environment, recent studies have unveiled the presence of a microbial community (Howland, 1998). The fundamental lung microbiota includes *Actinobacteria, Bacteroidetes, Firmicutes*, and *Proteobacteria* (Hou et al., 2022).

In a healthy state, the host’s immune response to the microbiota, in the gut and airway, is strictly compartmentalized to the respective mucosal surfaces. Many mechanisms are employed to achieve microbiota compartmentalization (Zheng et al., 2020b). These elements collectively contribute to the precision of immune responses, including barriers like TJs, AJs, mucus, and physiological components such as PRRs, lymphoid tissues, glycocalyx, NADPH oxidases, lactoperoxidase (LPO), neutrophil gelatinase-associated lipocalin (NGAL), secretory Immunoglobulin A, and the polymeric immunoglobulin receptor (PIGR). Collectively, these components contribute to the prevention of microbial colonization by exerting bacteriostatic effects, also regulate and localize the microbiota within specific anatomical compartments. This intricate compartmentalization of immune responses ensures a delicate balance between tolerance to benign microbes and defense against potential pathogens.

Intestinal epithelial cells serve as a crucial physical barrier, separating the host’s internal milieu from the gut luminal environment (Helander and Fändriks, 2014). Microbial signals, exemplified by the metabolite indole, actively contribute to reinforcing the epithelial barrier by upregulating tight junctions and associated cytoskeletal proteins (Bansal et al., 2010). TJs play a critical role in limiting trans-epithelial permeability, working in harmony with intracellular signaling and membrane-spanning proteins to maintain barrier integrity (Vaishnava et al., 2008; Soderholm and Pedicord, 2019).

Epithelial cells discern between pathogenic and commensal bacteria, regulating immune responses within the intestinal microenvironment. The diverse array of IECs, including enterocytes, stem cells, enteroendocrine cells, Paneth cells, goblet cells, M cells, and tuft cells, express a wide range of PRRs. PRR activation by microbes initiates immune responses to mediate intestinal homeostasis (Pott and Hornef, 2012). TLR2 controls mucosal inflammation by enhancing intestinal epithelial cell barrier function (Cario, 2008). Furthermore, Peterson et al. demonstrated that TLR-4 activation is a central factor in the breakdown of the intestinal barrier after burn injuries (Peterson et al., 2010).

Airway epithelial cells also express PRRs but are more involved in the defense against respiratory pathogens than in homeostasis (Hewitt and Lloyd, 2021).The lung microbiome undergoes dynamic changes influenced by factors such as microbial immigration (e.g. inhalation of bacteria), elimination (e.g. cough), and reproduction rates of its community members, as determined by regional growth conditions (e.g. nutrient availability) (Dickson et al., 2014).

While the airway epithelium predominantly induces antimicrobial products in response to TLR, the gut epithelium secretes pro-inflammatory cytokines such as IL-8. IL-18 secretion influences mucus production and composition, connecting Toll-like receptor-dependent cytokine production by intestinal epithelial cells to the presence of M cells, Paneth cells, and mucus-producing goblet cells within the epithelial tissue (Mcclure and Massari, 2014).

The mucus layer serves as a robust defense against infection. It fosters immune tolerance by preventing inflammation from beneficial gut microbes and supports a commensal gut and lung microbiome. A dense mucus layer forms a separation barrier between intestinal epithelium and resident microbes. The mucus barrier, organized around the hyperglycosylated mucin MUC2, provides protection through static shielding. MUC2 also limits the immunogenicity of intestinal antigens, influencing enteric dendritic cells towards an anti-inflammatory state. Intestinal DCs play a crucial role in compartmentalizing the enteric microbiota, involving mechanisms such as sampling gut bacteria for antigen presentation (Zhang and Wu, 2020).

Mucus in the respiratory tract, like in the gut, defends against infection. In healthy airways, MUC5B remains the dominant secretory mucin in submucosal glands and superficial airway epithelia, while MUC5AC is predominantly produced in superficial epithelia lining the proximal (cartilaginous) airways (Meldrum and Chotirmall, 2021).

The intestinal epithelial glycocalyx, consisting of glycosylated transmembrane mucins, serves as a crucial interface between the host and microbes. This protective layer is indispensable for nutrient absorption and when disrupted, has been implicated in various gastrointestinal diseases. The glycocalyx is a layer of glycoproteins on the surface of epithelial cells throughout the intestinal tract, protecting the intestinal mucosa from pathogens and mechanical stresses (Gayer and Basson, 2009). In the small intestine, glycocalyx covering epithelial cells provides attachment sites for normal flora, limiting pathogen colonization, and acting as a size-selective diffusion barrier. Beyond its protective functions, the glycocalyx contributes to mucosal lubrication, hydrophobicity, prevents auto-digestion and ulceration, participates in cellular signaling, and serves as a selective diffusion barrier for various substances. Impairment of the glycocalyx is associated with diseases such as inflammatory bowel disease and cancer, underscoring its critical role in intestinal function and homeostasis (Sun et al., 2020).

The alveolar epithelial glycocalyx, on the apical surface of alveolar epithelium, plays a crucial yet understudied role in lung homeostasis and injury. Distinct from the pulmonary endothelial glycocalyx, it is interposed between epithelial cells and surfactant. During injury, inflammatory stimuli activate proteases, leading to glycocalyx shedding. This shedding contributes to lung injury by causing alveolar hyperpermeability, disrupting surfactant function, enhancing bacterial virulence, and impairing epithelial cell repair (Rizzo et al., 2022; Rizzo and Schmidt, 2023).

The NADPH oxidase (Nox) and Dual oxidase (Duox) enzyme family, comprising seven members in mammals, play a vital role in the intricate interplay between epithelial cells and the microbiota. These enzymes catalyze the production of reactive oxygen species (ROS), including superoxide and hydrogen peroxide, in response to microbial challenges (van der Vliet et al., 2018). Specifically, DUOX2/DUOXA2 serve as the primary hydrogen peroxide (H_2_O_2_) producing system in the human colorectal mucosa and ileum (highest expression), releasing substantial quantities of H_2_O_2_ from the epithelial layer into the gut lumen as part of the innate immune response (Macfie et al., 2014; Shu et al., 2023). The H_2_O_2_ produced by epithelial cells acts as a defense mechanism, limiting microbial colonization and exhibiting toxicity against pathogens. H_2_O_2_ is toxic to pathogens and aids in crosslinking mucus, enhancing viscosity and reducing permeability to bacteria (Németh et al., 2007; Carlson et al., 2018). Certain bacteria, such as E*nterobacteriaceae*, deploy catalase to neutralize H_2_O_2_ and evade the host’s immune response (Pericone et al., 2000; Macfie et al., 2014; Sommer and Bäckhed, 2015).

The interaction of thiocyanate (SCN−), mainly derived from dietary sources, with H_2_O_2_, catalyzed by LPO, forms the biocide hypothiocyanite (OSCN^-^), which is effective against a wide range of microorganisms (Magacz et al., 2019). The DUOX2/DUOXA2 system is upregulated during bacterial infection (Shu et al., 2023). DUOX1/2 in tracheal epithelial cells produce H_2_O_2_, inducing an antimicrobial system. DUOX1 activation drives mucin secretion, suggesting therapeutic potential in inflammatory airway diseases. NOX/DUOX isoforms contribute to host defense, innate immunity, and mucosal healing, with potential implications in lung diseases. Notably, the airway secretion’s LPO system has demonstrated antibacterial activity against *Pseudomonas aeruginosa*, *Burkholderia cepacia*, and *Haemophilus influenzae*. This functional LPO system in the airways may contribute significantly to airway host defense against infections (Bernard et al., 2014).

NGAL, also referred to as lipocalin-2, binds to bacterial ferric siderophores, which are essential for the uptake of iron by bacteria (Wijkstrom-Frei et al., 2003). NGAL plays a crucial role in protecting against bacterial infection and modulating oxidative stress in normal tissues. Functioning as a potent bacteriostatic agent under iron-limiting conditions, NGAL represents a unique iron-depleting antimicrobial defense strategy. It is secreted in small amounts by immune cells (neutrophils and macrophages), epithelial cells, smooth muscle cells, hepatocytes, adipocytes, and neurons, and its levels can be measured in serum or feces under physiological conditions chakraborty (Cowland et al., 2003; Chakraborty et al., 2012).

Elevated NGAL concentrations in serum are associated with injury to epithelial cells in the gastrointestinal and respiratory tracts. NGAL is highly expressed by epithelial tissues, especially in the lung and trachea, and upregulated in response to inflammation (Cowland et al., 2003; Chakraborty et al., 2012).

The mucosal immune system orchestrates a dynamic interplay between epithelium and microbiota, particularly facilitated by secretory immunoglobulin A (SIGA). Innate lymphoid cells contribute to rapid cytokine secretion, combatting infection, and promoting mucosal tissue repair. B cells produce SIGA responsive to commensals (Zheng et al., 2020b). The mutualistic relationship between intestinal IGA and microbiota involves the diversified IGA repertoire maintaining a balanced microbiome. Colitogenic bacteria are preferentially coated by IGA, preventing perturbations and inflammation. In the absence of B cells or IGA, epithelial immune defense mechanisms are upregulated, altering microbiome composition, and impacting metabolic functions (Bunker and Bendelac, 2018; Zheng et al., 2020b).

SIGA is transported across epithelium by the polymeric immunoglobulin receptor (PIGR). Expressed by various cells, IIGR facilitates the transcytosis of dimeric IGA (DIGA). SIGA’s crucial roles include immune exclusion, neutralizing antigens, and regulating immune cells and microbiota interactions. In the airway, alterations in the PIGR/IGA system are observed in respiratory diseases. Overall, SIGA acts as a vital component of the mucosal immune system, contributing to immune defense, symbiosis with microbiota, and mucosal homeostasis (Bunker and Bendelac, 2018; Zheng et al., 2020b).

Pathogenic infections and infection-induced microbial dysbiosis can compromise the integrity of the intestinal barrier, resulting in bacterial translocation from the intestine. Under pathological conditions, tight junctions and adherens junctions may be disrupted, providing pathways for bacteria, bacterial LPS, toxins, and enzymes to breach the intestinal barrier through paracellular routes, facilitating bacterial translocation (Pott and Hornef, 2012).

### Resident immune cells: orchestrating epithelial defense and tissue homeostasis

IPRRs and AMPs connect innate immune defenses with intricate signaling pathways.

A network of local immune surveillance systems, coordinated by macrophages, dendritic cells (DCs) and adaptive and regulator T cells monitor epithelial health and integrity (Suresh and Mosser, 2013; Fan and Rudensky, 2016). Adaptive T cells in general are characterized by markers such as CD3, CD4, CD8, CD45RO, CD62L, CD45RA, and CD25 (Golubovskaya and Wu, 2016; Mangare et al., 2019). Regulatory T cells are identified by a panel of well-established markers, including CD4, CD25, FOXP3, CD103, CCR9, CD69, α4β7 integrin, GPR15 (LPAM-1), and CD44, which are associated with their development, function, tissue residency, and migratory properties (Liston and Gray, 2014; Lu et al., 2015; Richards et al., 2015; Jacobse et al., 2021). DCs are marked by XCR1, SIRPα (CD172a), CD1c, CD16 (FCγRIIIA), BDCA-3 (CD141) and subdivided into DC1 and DC2 by expression (Mcdole et al., 2012; Lombardi and Khaiboullina, 2014; Stagg, 2018).

Progenitor cells respond to immune challenges by communicating with the resident immune sentinels. Responding immune effectors enter from circulation to infiltrate stressed tissues, clear invading pathogens, activate repair processes, and reinstate homeostasis (Naik et al., 2018).

The landscape of resident immune cells differs across tissues, contributing to unique tissue function. Epithelial tissues in skin, lung, and gut are noted for their heightened immune activity, navigating the challenges posed by environmental stressors, both physical and pathogenic (Niec et al., 2021).

Recent years have witnessed a paradigm shift in our understanding of immune cells, extending beyond their canonical roles in pathogen defense. There is new evidence resident immune cells contribute to the homeostatic regulation of progenitors. Macrophages and regulatory T cells (Tregs) have specifically been identified as regulators of stem cells under normal physiological conditions (Burzyn et al., 2013; Davies et al., 2013; Naik et al., 2018).

The composition and abundance of resident immune cells within tissues is shaped resident cells and the inflammatory milieu. As epithelial barrier tissues, the lungs and gut host a diverse array of immune players, including dendritic cells, macrophages, ILC subsets, γδ T cells, and Tregs, which seed these tissues early in life. Over time, exposure to commensals and pathogens enriches the tissue with CD8^+^T resident memory cells (T_RM_) and circulating CD4^+^T helper subsets, a process documented by Barber et al. (2006).

Lymphoid tissues (like Peyer’s patches and nasal-associated lymphoid tissue) house resident immune cells, crucial for ongoing immune function in homeostasis. Their activity intensifies during stress, rapidly mounting effective immune responses to bolster the body’s defense mechanisms (Iwasaki and Kelsall, 2000).

Peyer’s patches, initially identified in the human small intestine by Cornes in 1965, are observed in mammals, including humans, and serve as vital immunosensors. Peyer’s patches manage luminal antigens and bacteria. Comprised of follicle-associated epithelium and a resident immune cell population (B cells, T cells, dendritic cells) within the lymphoid follicles, Peyer’s patches form a luminal barrier facilitated by specialized epithelial microfold cells crucial for the sampling and transport of antigens.

Immune responses in Peyer’s patches are regulated by pathogen recognition receptors such as NOD2, which impact T cell characteristics and epithelial permeability (Peterson and Artis, 2014; Haddad et al., 2023). The journey of luminal antigens involves transportation from the mucosal surface of Peyer’s patches to the subepithelial dome (SED), facilitated by the specialized epithelial M cells of the follicle-associated epithelium. Within the SED, resident DCs play a crucial role in antigen uptake, with some expressing elevated levels of lysozyme (LysoDC) and exhibiting strong phagocytic activity (Iwasaki and Kelsall, 2000; Lelouard et al., 2010).

The respiratory tract houses a diverse array of lymphoid tissues, including tonsils, adenoids, nasal-associated lymphoid tissue (NALT), and bronchus-associated lymphoid tissue (BALT), alongside lymph nodes responsible for upper and lower respiratory tract drainage.

In response to acute stress, an orchestrated recruitment of inflammatory macrophages/monocytes, neutrophils, basophils, and eosinophils occurs at the site of damage. Recruited immune cells bolster function of resident immune cells, enhancing the tissue’s capacity to manage stressors effectively. Lymphoid organs, such as the lymph nodes and spleen, function as epicenters for naive or unprimed T cells. Primed by dendritic cells, T cells differentiate into effectors before migrating into the affected tissue, where they exert their specific immune functions, such as cytokine release, cytotoxicity, or coordination of the immune response. Interestingly, granulocytes, including neutrophils, traditionally reserved for active immune responses, are not conventionally categorized as tissue resident in non-lymphoid tissues under steady-state conditions (Sakaguchi et al., 2013; Naik et al., 2018).

Macrophages, present throughout the body, exhibit considerable functional diversity influenced by their origin and the inflammatory conditions of their surroundings (Epelman et al., 2014). The term ‘macrophage’ encompasses various cell types, renowned for their proficiency in engulfing deceased cells. They were among the earliest identified immune cells known for their ability to regulate stem cells. Macrophages were recently identified as key components of ISC crypts, which are responsible for sustaining continuous production of the TA cells and their differentiated epithelial cell progeny (including goblet cells, enterocytes, EECs, and Tuft cells) (Tan and Barker, 2014).

Alveolar macrophages are specialized macrophages of the lung that demonstrate noteworthy diversity in their origin, molecular characteristics, and functions (Misharin et al., 2013; Tan and Barker, 2014). A compelling study by Engler et al. (2020) revealed that certain immune cells and macrophages facilitate lung recovery after partial removal in a mouse model, shedding light on their role in tissue repair (Naik et al., 2017, Naik et al., 2018; Engler et al., 2020).

Tregs, characterized by the expression of the transcription factor FOXP3, constitute a subset of CD4+ helper T lymphocytes primarily harbored in the bone marrow. Similar to macrophages, Tregs exhibit functional diversity and plasticity, adapting to different physiological states (Sakaguchi et al., 2013). Renowned for their potent immunosuppressive capabilities, Tregs play a crucial role in preventing harmful autoimmunity (Josefowicz et al., 2012).

Investigations into stem cell niches, often characterized by low inflammatory cell presence, prompt speculation about Tregs potentially residing in these immune-privileged sites (Fujisaki et al., 2011; Hirata et al., 2018). Tregs emerge as key guardians of the integrity of LGR5+ intestinal stem cells, with their depletion resulting in a significant reduction in ISC numbers. LGR5, also known as Leucine-rich repeat-containing G protein-coupled receptor 5, is a cell surface protein that serves as a marker for ISCs. Co-culturing intestinal organoid cultures with Tregs or their effector cytokine IL-10 demonstrates a notable enrichment of LGR5+ISCs, reinforcing the direct influence of Tregs on stem cells (Biton et al., 2018; Naik et al., 2018).

**Figure 5:**
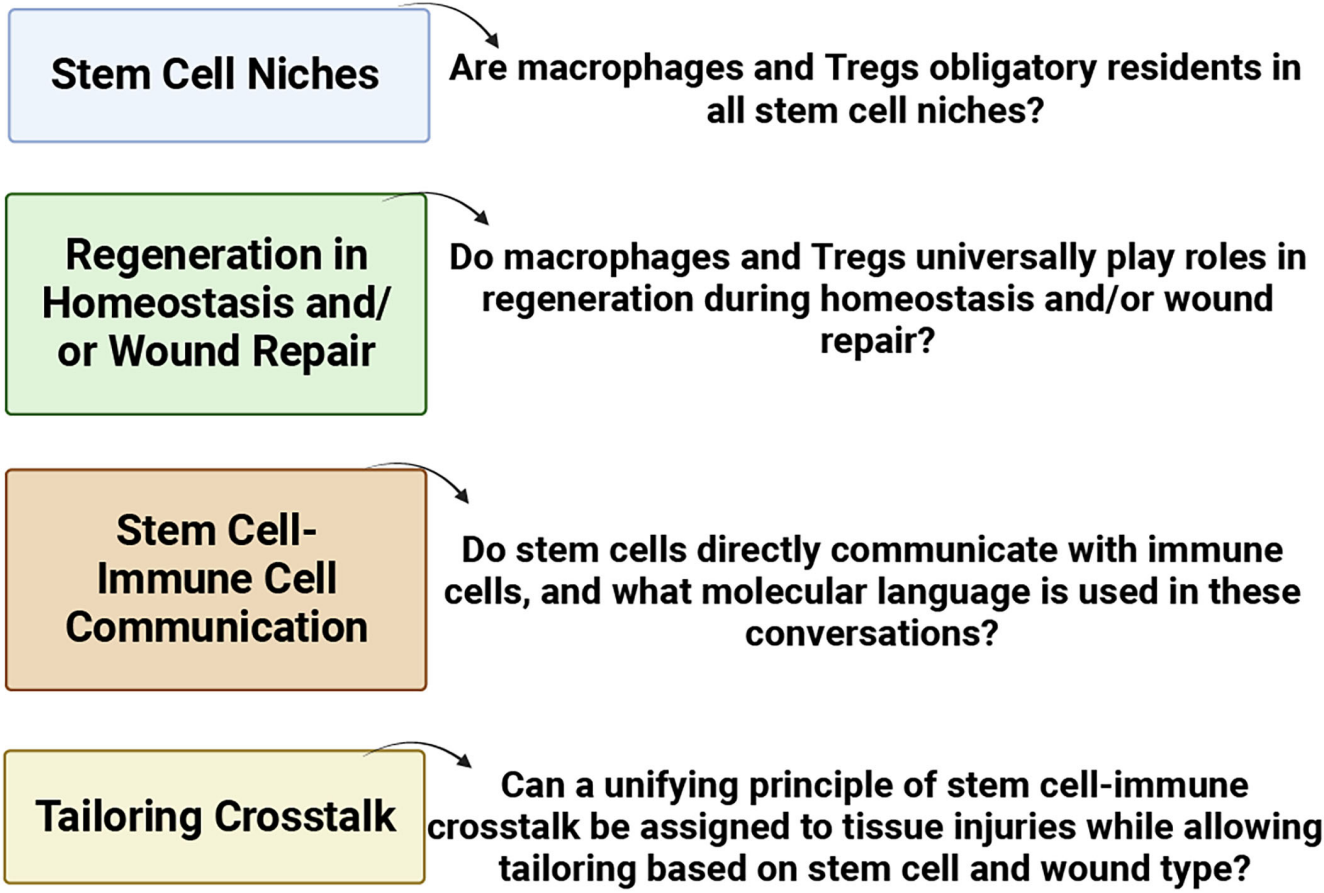
Pressing issues in stem cell-immune crosstalk in tissue regeneration (BioRender, 2023).

The involvement of Tregs in respiratory diseases, such as allergic rhinitis and chronic rhinosinusitis, underscores their role in modulating immune profiles. Diseases characterized by a skewed type 2 immune response often coincide with reduced Treg presence (Palmer et al., 2017; Naik et al., 2018). Recognizing the pivotal role of Tregs in controlling inflammation, attention has turned to their potential contribution to tissue regeneration. This is particularly relevant in the context of macrophages and Tregs, both of which appear to dualistically modulate immunity and promote regeneration. Numerous questions persist (as highlighted in **Figure 5**) concerning stem cell niches, regeneration in homeostasis and/or wound repair, stem cell-immune cell communication, and tailoring crosstalk. The prospect of identifying unified principles governing stem-cell-immune crosstalk in tissue injuries becomes an intriguing avenue for exploration (Gersemann et al., 2011; Naik et al., 2017; Ordovas-Montanes et al., 2018).

### Immune cells and stem cells: from tissue repair dynamics to advanced models of epithelial diseases and beyond

The historical discourse between immune cells and stem cells during the process of tissue repair is both intriguing and vital. Stem cells, acting as proficient repair agents, assume a pivotal role in mitigating the repercussions of tissue damage. Analogously, they serve as an initial alarm system, signaling the need for assistance, possibly orchestrating the immune response. The collaboration between immune cells and local progenitors could significantly expedite the healing process. Research endeavors delve into elucidating this intricate relationship, particularly within tissues exposed to heightened microbial threats and frequent damage (Naik et al., 2017, Naik et al., 2018; Engler et al., 2020).

Specific immunological modules, notably T helper (Th) 17, 1, and 2 responses, come into play in instances of prolonged immune challenges. For instance, the Th17 response orchestrates the recruitment of immune cells to eliminate extracellular pathogens, while the Th1 response activates macrophages to combat intracellular threats. Simultaneously, Th2 responses play a pivotal role in addressing parasitic infections (Vahedi et al., 2013). Stem cells, finely attuned to these dynamic environments, demonstrate adaptability in behavior, reinforcing tissue integrity and contributing substantively to host defense.

Consider a scenario involving a parasitic infection. The Th2 cytokine IL-13 assumes a central role, signaling intestinal stem cells to undergo differentiation. This prompts ISCs to contribute to the expulsion of parasites by synthesizing mucus, simultaneously fortifying Th2 responses (von Moltke et al., 2016). During an active immune response, immune effectors congregate around stem cells, creating a specialized microenvironment termed the “inflamed niche”, thereby influencing stem cell behavior (Naik et al., 2018). In the airway, IL-13 promotes mucus synthesis, regulates Immunoglobulin E production, and orchestrates cytokine production by macrophages (Glisinski et al., 2020).

The mechanics that drive stem cells to resume homeostatic functions after resolution of an inflammatory event remain elusive. However, failure to achieve resolution of an active immune response may culminate in chronic inflammation, adversely affecting various tissue progenitors in conditions such as psoriasis, atopic dermatitis, asthma, rhinosinusitis, and IBD (Naik et al., 2018).

#### Epithelial stem cells and their role in tissue homeostasis and regeneration

Epithelial stem cells are integral to the maintenance of tissue homeostasis and the facilitation of wound repair. Their fate and function are precisely regulated by the microenvironment in which they reside. Notch signaling, a key pathway akin to WNT signaling, plays a central role in dictating cell fate across various tissues (Artavanis-Tsakonas et al., 1999).

To ensure lifelong tissue homeostasis, stem cells must self-renew continuously while giving rise to differentiated progeny. This delicate balance, where at least one daughter cell retains stem cell properties after each division, is under the influence of the Wnt/β-catenin signaling pathway. Dysregulation of this pathway is frequently associated with the development of familial and sporadic epithelial cancers (Reya and Clevers, 2005; Mazemondet et al., 2012). The Wnt/β-catenin pathway, an ancient and evolutionarily conserved mechanism, plays a pivotal role in the orchestration of development and morphogenesis across diverse tissues (Logan and Nusse, 2004). In mammals, approximately 20 WNT proteins, secreted cysteine-rich molecules, contribute to this regulatory network (Willert and Nusse, 2012).

The canonical Notch signaling pathway is highly conserved and guides short-range cell-cell interactions (Kopan and Ilagan, 2009). In mammals, four transmembrane Notch receptors (Notch1-4) and Notch ligands, transmembrane proteins categorized into three subfamilies, play indispensable roles in various tissues. Notch signaling is known to have dual effects on epithelial stem cells, promoting maintenance and self-renewal in some contexts while inhibiting these processes in others (Guo et al., 2011). Disruption of canonical Notch signaling in embryonic epidermis, for example, results in reduced proliferation and impaired differentiation (Blanpain et al., 2006). It remains an area of ongoing research to determine whether the observed hyperproliferation following Notch1 ablation is linked to noncanonical Notch signaling alterations or indirectly results from epidermal barrier defects (Blanpain et al., 2006). The Bone Morphogenetic Protein (BMP) signaling pathway influences the activation of several types of epithelial stem cells (Blanpain et al., 2007; Bond et al., 2012) but the role of additional signaling pathways in epithelial stem cell biology are less clear. SHH, for instance, exerts a significant impact on hair follicle morphogenesis but has varying effects on the development of different epithelial tissues (Carballo et al., 2018). Aberrations in SHH signaling or its negative regulator, Patched, have been linked to various epithelial cancers, including basal cell carcinomas and pancreatic carcinoma (Jeng et al., 2020). Interestingly, reducing hedgehog signaling seems to enhance cell proliferation in some epithelial cell types while inhibiting or not affecting proliferation in others (Blanpain et al., 2007).

The exploration of mechanisms governing epithelial stem cell behavior is a rapidly advancing field with increasing biomedical significance for developing treatments for damaged or cancerous epithelial tissues. Scientists, clinicians, and patients alike anticipate further discoveries in this captivating realm of epithelial stem cells (Blanpain et al., 2007).

#### Cellular events in tissue repair

Addressing the intricate realm of epithelial biology involves an exploration extending from understanding the pivotal role of epithelial stem cells and their contributions to tissue homeostasis and regeneration. Building upon this foundation, a seamless transition leads into a comprehensive examination of cellular events in tissue repair. These interconnected topics unravel the dynamic processes that govern epithelial responses, offering a holistic view of the intricate mechanisms underlying both tissue homeostasis and the reparative phases.

Immune cells and stem cells collaborate through a series of cellular events to repair tissue damage. The restoration of epithelial surface continuity involves a multifaceted response to extensive damage. Initially, neighboring epithelial cells migrate to the injured site, effectively covering the denuded area. Subsequently, the proliferation of epithelial cells becomes imperative for replenishing the diminished cell pool. Finally, the maturation and differentiation of undifferentiated epithelial cells are essential to maintain the diverse functional activities of the mucosal epithelium (Zhang et al., 2014).

Following injury, the initial response involves an acute inflammatory reaction, attracting immune cells and facilitating the spreading and migration of epithelial cells. This coordinated migration is guided by autologously secreted provisional matrices and influenced by DAMPs, playing a pivotal role in the overall tissue repair process (Croasdell Lucchini et al., 2021; Marconi et al., 2021; Frede et al., 2022).

In the phase of cell proliferation triggered by injury, essential factors are released by the damaged or injured tissue, including growth factors (TGF-, KGF, HGF), chemokines (MCP-1), interleukins (IL-1, IL-2, IL-4, IL-13), and prostaglandins (PGE2). These factors orchestrate complex processes involving integrins, matrix materials, matrix metalloproteinases, focal adhesions, and cytoskeletal structures, ultimately promoting the proliferation of epithelial cells and contributing to the regeneration of damaged tissue (Crosby and Waters, 2010; Iizuka and Konno, 2011; Zhang et al., 2014).

Cell differentiation, another critical aspect of tissue repair, is regulated by various signaling pathways such as SHH, Rho GTPases, MAP kinase pathways, STAT3, TGF-β, WNT, and TNF-α. Proinflammatory factors like TNF-α play a role in promoting mucosal wound repair by activating the WNT/β-catenin signaling pathway to enhance epithelial cell proliferation and upregulate receptors that facilitate intestinal healing. Recruitment of localized and distal progenitor stem cells to the injured area contributes to the recovery of epithelial function (Crosby and Waters, 2010; Iizuka and Konno, 2011; Zhang et al., 2014).

#### Advanced models of epithelial diseases

The proper function of vital organs relies on a functional epithelium. Efforts in tissue engineering have focused on replicating epithelial tissues in laboratory settings, yielding promising results in constructing organs like the bladder and trachea. As regenerative medicine advances, the creation of complex microscale tissue models for drug discovery and disease simulation is set to expand, aiding both basic research and clinical applications (Vrana et al., 2013).

Animal models play a crucial role in replicating human diseases and advancing pharmaceutical drug development. Mice, for instance, can be genetically modified and humanized, creating a dynamic environment that mirrors the complexity of human organs. Organs do not operate in isolation, and the circulation of nutrients, hormones, and mediators throughout the body adds to this complexity. This enables real-time analysis of drug-organ interactions, especially in absorption, distribution, metabolism, and excretion. Despite the dominance of murine models, potential limitations, including difficulty in direct translation to human scenarios, should be acknowledged (Blum et al., 2015; Garattini and Grignaschi, 2017).

Species differences exist in airway and gut physiology. In the airway, variations in lung size, branching patterns, and cellular components exist among species, necessitating multi-strain testing for a comprehensive understanding of drug impact. In the gut, mice are commonly used to study microbial diversity, but differences in size, mucosal structure, and feeding patterns between mice and humans should be considered during experimental design. Notably, mice exhibit variations in intestinal pH, oxygen tension, and glycan profile compared to humans, influencing microbial composition (Karra et al., 2018; Hewitt and Lloyd, 2021).

In cell culture, cell lines are categorized as either monolayer or suspension types, each exhibiting a unique doubling time influenced by the cell cycle. Cultured cells progress through distinct phases: lag, log (characterized by exponential growth), plateau, and decline. When it comes to simulating organs and creating artificial tissues, specific cell lines play crucial roles. For mimicking lung conditions, widely utilized cell lines include 16HBE, A549, Calu-3, and NHBE. Similarly, for modeling the intestinal environment, established human cell lines like Caco-2 or HT-29 are commonly employed (Langerholc et al., 2011; Nikolic et al., 2018).

Organoids, self-organized 3D tissues typically derived from various stem cell sources (pluripotent, fetal, or adult), intricately replicate the functional, structural, and biological complexity of organs, serving as microscale models for the study of diseases and drug responses (Huch and Koo, 2015).

In contrast to traditional cell lines, organoids present a more authentic representation of *in vivo* tissues, showcasing heightened complexity by incorporating diverse cell types and intricate structures. This nuanced composition contributes to a more accurate portrayal of human organ physiology. Depending on the targeted organ, organoids can comprise various cell types, such as epithelial cells, goblet cells, and enterocytes, enabling the formation of complex structures reminiscent of natural organs (Antfolk and Jensen, 2020; Kim et al., 2020; Zhao et al., 2022).

Activation of WNT and FGF pathways is imperative for the growth, development, and maintenance of organoids, while the suppression of BMP and TGF signaling is crucial to prevent hindrances in cell growth and differentiation across various organoid origins (Antfolk and Jensen, 2020; Kim et al., 2020). Organoids can be cultivated either embedded in extracellular matrices (ECMs) or in suspension, with the choice guided by specific research goals and the desired microenvironment for the organoid (Zhao et al., 2022).

The advent of human organoids, engineered from genetically modified stem cells or patient biopsy samples, offers detailed replication of organ architecture. These models, provide unique opportunities for disease study, complementing animal models. Despite challenges like microenvironmental gaps and higher costs, human organoids significantly contribute to advancing our understanding of human physiology and disease (Antfolk and Jensen, 2020; Kim et al., 2020). For certain applications, such as drug testing and disease modeling, transitioning from three-dimensional organoids to two dimensional monolayers can be advantageous. The process involves dissociating the organoids into single cells and allowing them to adhere and grow as a monolayer on a flat surface, such as a culture dish. This transition to a two-dimensional format simplifies experimental procedures and enables high-throughput screening (van Dooremalen et al., 2021).

Organ on a chip system involve the cultivation of engineered or natural miniature tissues within microfluidic chips, designed to intricately control cell microenvironments and uphold tissue-specific functions. The microfluidic devices, resembling a network of hair-fine microchannels, guide and manipulate minute solution volumes ranging from picoliters to millilitrers. The term ‘organ’ in this context pertains to the miniature tissues cultivated within these microfluidic chips. Essential growth factors, including IGF, EGF, FGF, and TGF, are supplied to sustain cellular health and function (Karra et al., 2018; Nikolic et al., 2018).

Like organoids, organ on a chip device offer a nuanced representation of *in vivo* organs, surpassing the capabilities of traditional cell lines. The microfluidic design fosters dynamic interactions between cells and their environment, providing a closer approximation to the complexity of human organs (Leung et al., 2022).

*In vitro* models are tailored to replicate the structures of specific organs, integrating relevant cell types. For instance, lung on a chip device may incorporate epithelial cells, immune cells, and endothelial cells to simulate the alveolar environment. Primarily designed for adherence-based cultures, organ on a chip device typically rely on extracellular matrices (ECMs) to provide a substrate for cell attachment (Karra et al., 2018; Nikolic et al., 2018).

Organs on a chip address challenges in recapitulating human diseases through *in vitro* cell culture. Lung on a chip device mimic *in vivo* conditions, and guts-on-chip platforms have evolved to include intricate functionalities. The strength of organs-on-a-chip lie in faithfully replicating structural and functional complexities, surpassing traditional *in vitro* techniques. Challenges include concerns about reproducibility and robustness, necessitating standardization for commercialization (Karra et al., 2018; Nikolic et al., 2018).

Physiologically relevant *in vitro* models cultured at the air-liquid interface (ALI) are becoming efficient tools for lung toxicity testing and cell-cell interaction studies. Primary bronchial epithelial cells cultured at ALI differentiate into a respiratory epithelium with multiple cell types. Advanced bronchial ALI models feature fully differentiated epithelia with immunocompetent cells. This setup promotes the differentiation of epithelial cells, leading to structures and functions resembling *in vivo* counterparts. Moreover, it is possible to culture intestinal explants containing both epithelial and mesenchymal cells into spheroid-like organoids using an ALI methodology that does not require exogenous growth factor supplementation (Li et al., 2016).

ALI cultures are essential for studying airway biology, including responses to inhaled substances, pathogens, and diseases. This system is vital for drug testing, toxicity studies, assessing barrier functions, and understanding diseases involving epithelial barrier dysfunction. ALI cultures also facilitate the study of host-pathogen interactions in respiratory infections, providing a versatile tool for diverse research areas (Upadhyay and Palmberg, 2018, 2018; Leach et al., 2023).

Traditionally, 2D cell cultures were extensively employed in disease studies but exhibited limitations. Transitioning toward 3D models using specialized structures like 3D scaffolds offers environments more like the human body, facilitating improved cell interactions crucial for understanding diseases and drug responses. However, working with 3D cell cultures presents challenges in ensuring proper nutrient distribution, crucial for refining these models to better replicate human tissues (Loh and Choong, 2013; Duval et al., 2017; Creff et al., 2021). Understanding the advantages and limitations of these techniques guides future research, aiming for more realistic models replicating human organs.

## Conclusion

In conclusion, the literature review has offered a comprehensive understanding of the intricate relationships and functions within internal epithelial cells at the interface with the outside world, with a particular focus on the gut and airway epithelia. Diving into various signaling pathways and mechanisms, the review illuminates how epithelial cells maintain homeostasis and engage with immune cells, stroma, and progenitors.

The gastrointestinal and airway epithelial layers serve as the frontline defense, producing an array of antimicrobial substances and facilitating swift paracrine signaling among epithelial cells in response to microbial encounters. The airway, tasked with intricate functions such as gas exchange, surfactant secretion, and the operation of a mucociliary escalator comprising mucus-secreting goblet cells and beating ciliated cells, effectively clears debris from the airways. In contrast, the gastrointestinal tract, characterized by potentially deleterious chemical processes, maintains the integrity of its absorptive epithelium through constant and rapid renewal. Specifically, the gut epithelium, exposed to diverse microorganisms via the oral route, has evolved to establish a symbiotic relationship with commensal bacteria. This stands in sharp contrast to the respiratory epithelium, which confronts the persistent challenge of filtering and defending against airborne pathogens and pollutants.

While research on intestinal epithelial morphogenesis and regeneration is progressing rapidly, there exists a crucial need for additional studies to delve deeper into the nuances of these processes. Discrepancies and challenges, such as understanding interspecies differences between mouse and human epithelium, warrant further exploration.

The emergence of advanced *in vitro* lung models, including lung organoids and air-liquid interphase cultures, holds promise for enhancing our understanding of human airway plasticity during development, homeostasis, and disease.

In essence, the literature review provides valuable insights into the dynamic world of epithelial cells, showcasing their interactions with the immune system and highlighting the potential for therapeutic advancements in regenerative medicine. The multifaceted nature of epithelial responses, spanning from intricate signaling pathways to adaptive cell plasticity, underscores the significance of ongoing research in unraveling the complexities of epithelial biology.

### Limitations

While this literature review aimed to provide insight into the intricate interplay between immune signaling pathways and epithelial homeostasis, there are certain limitations that should be acknowledged. Firstly, due to the breadth of the topics covered, including pathogen detection mechanisms, antimicrobial effectors, and immune cell interactions, it was challenging to delve into each area in extensive detail within the confines of this review.

Furthermore, the complexity of epithelial-immune interactions and the rapidly evolving nature of research in this field pose challenges in achieving an exhaustive analysis. While we aimed to highlight key findings and emerging trends, there may be nuances and recent advancements that were not fully addressed in this review.

## Author contributions

MA: Writing – original draft, Conceptualization. CV: Writing – review & editing.
